# Diethyldithiocarbamate-copper complex (CuET) inhibits colorectal cancer progression via miR-16-5p and 15b-5p/ALDH1A3/PKM2 axis-mediated aerobic glycolysis pathway

**DOI:** 10.1038/s41389-020-00295-7

**Published:** 2021-01-08

**Authors:** Xin Huang, Yichao Hou, Xiaoling Weng, Wenjing Pang, Lidan Hou, Yu Liang, Yu Wang, Leilei Du, Tianqi Wu, Mengfei Yao, Jianhua Wang, Xiangjun Meng

**Affiliations:** 1 Department of Gastroenterology, Shanghai Ninth People’s Hospital, Shanghai Jiao Tong University School of Medicine, 200011 Shanghai, China; 2 Digestive Disease Research and Clinical Translation Center, Shanghai Jiao Tong University, 200011 Shanghai, China; 3 Cancer Institute, Fudan University Shanghai Cancer Center, Fudan University, 200032 Shanghai, China

**Keywords:** Cancer genetics, Drug regulation, Cancer microenvironment

## Abstract

Exploring novel anticancer drugs to optimize the efficacy may provide a benefit for the treatment of colorectal cancer (CRC). Disulfiram (DSF), as an antialcoholism drug, is metabolized into diethyldithiocarbamate-copper complex (CuET) in vivo, which has been reported to exert the anticancer effects on various tumors in preclinical studies. However, little is known about whether CuET plays an anti-cancer role in CRC. In this study, we found that CuET had a marked effect on suppressing CRC progression both in vitro and in vivo by reducing glucose metabolism. Mechanistically, using RNA-seq analysis, we identified ALDH1A3 as a target gene of CuET, which promoted cell viability and the capacity of clonal formation and inhibited apoptosis in CRC cells. MicroRNA (miR)-16-5p and 15b-5p were shown to synergistically regulate ALDH1A3, which was negatively correlated with both of them and inversely correlated with the survival of CRC patients. Notably, using co-immunoprecipitation followed with mass spectrometry assays, we identified PKM2 as a direct downstream effector of ALDH1A3 that stabilized PKM2 by reducing ubiquitination. Taken together, we disclose that CuET treatment plays an active role in inhibiting CRC progression via miR-16-5p and 15b-5p/ALDH1A3/PKM2 axis–mediated aerobic glycolysis pathway.

## Introduction

Colorectal cancer (CRC) is one of the most commonly diagnosed cancers among adults in many countries. It is the second leading cause of cancer mortality, with more than 50,000 death tolls per year^1^. Notably, CRC is one of the most prevalent neoplasm in the male population, with a rapid increase in both incidence and mortality recently^2^. Although systematic therapeutic approaches including chemotherapy and immunotherapy have reduced cancer-specific mortality, CRC still has a higher incidence of cancer deaths due to metastasis. Therefore, new drugs are warranted to effectively inhibit proliferation and promote apoptosis of CRC cells, slow down the progression of CRC, and improve clinical prognosis.

Disulfiram (DSF) is a Food and Drug Administration (FDA) approved anti-alcoholism drug since the 1940s^3^. In the past decade, in vitro and in vivo studies have shown that DSF has great potential in cancer treatment such as lung cancer^4,5^, liver cancer^6–8^, and breast cancer^9–11^. Furthermore, it has been found that DSF is metabolized to diethyldithiocarbamate (ditiocarb, DTC) in vivo and forms complex with copper. It is DTC-copper complex (CuET) that plays the ultimate role in anti-cancer^12,13^. However, the role and mechanisms of CuET in inhibiting CRC progression still remain largely unknown.

Tumor cells consume a large amount of glucose to produce lactate, despite ample oxygen supply. This phenomenon is called aerobic glycolysis, or the “Warburg effect”, a unique hallmark of metabolic mechanism to fuel cancer cells with energy^14^. Recently, new approaches such as gene therapy, including genetic/epigenetic aberrations intervention, immune gene therapy, suicide gene therapy, and recently developed CRISPR-mediated PD-1 gene knockout technology were used to treat CRC^15–17^. However, it is challenging to identify patients who respond to the treatment because the complexity of tumor microenvironment is largely ignored. Studies have shown that targeting the expression of metabolic enzymes such as HK-II, PFK, GAPDH, PKM2, PDH, and LDH can affect tumor development^18–20^. These results suggested tumor-specific aerobic glycolysis might be the potential therapeutic target for tumor therapy. Recently, accumulated research data showed that the aerobic glycolysis in CRC has changed. For example, downregulation of Myc-related metabolic enzymes causes the decrease of glycolysis, which accounts for the arrest of proliferation^21^; tumor-secreted DKK2 stimulates angiogenesis by promoting aerobic glycolysis^22^.

Notably, we found CuET can suppress tumor progression and reduce glucose metabolism. RNA-seq analysis identified ALDH1A3, an important isoform of the aldehyde metabolic enzyme system ALDH family, as a target gene of CuET. So far, ALDH1A3 has been found to be involved in the regulation of the biological characteristics of tumor cells, such as stemness, proliferation, invasion, metastasis, and drug resistance^23,24^. Over-expression of ALDH1A3 is closely associated with the initiation, progression, drug resistance, and poor prognosis of various tumors^25–27^. Thus, ALDH1A3 can be a potential marker for cancer diagnosis and therapeutic target, but the mechanism of action remains to be illustrated.

In this study, we identified that ALDH1A3 could interact with PKM2 to modulate tumor aerobic glycolysis regulated by its upstream miR-16-5p and 15b-5p upon CuET treatment in CRC.

## Materials and methods

### Clinical specimen

A total of 42 frozen CRC tissues and adjacent non-cancer tissues from patients undergoing surgery in the Ninth People’s Hospital Affiliated to Shanghai Jiao Tong University School of Medicine between 2013 and 2017 were analyzed (Jiuyuan cohort). All of these patients had not received chemotherapy or radiotherapy before the operation. Each patient signed a written informed consent form and ethical consent in accordance with the Ethical Committee of the Ninth People’s Hospital Affiliated to Shanghai Jiao Tong University School of Medicine. The clinical data of the CRC patients are shown in Supplementary Table 1.

### Mouse subcutaneous tumor xenograft experiments

A total of 5 × 10^6^ HCT116 cells diluted in 100 μl PBS were injected subcutaneously into the right flanks of 4-5 weeks old male nude mice obtained from Shanghai SLAC Laboratory Animal Co., Ltd. Mice were randomly assigned into three groups (*n* = 5/group): DMSO group and two CuET treatment groups. When tumor nodules were about 3-5 mm in diameter and became visible (12 days), CuET with different concentrations (25 mg/kg or 50 mg/kg) was intraperitoneally injected. Control mice received DMSO alone. The size of the tumor was measured with a scale every other day. After 21 days of administration, the nude mice were killed, photographed, and the tumor was removed, weighed, and recorded. The calculation formula of tumor volume is: tumor volume [mm^3^] = (length × width^2^). The mice experiment was carried out in strict accordance with the Ethical Committee of the Ninth People’s Hospital Affiliated to the Medical College of Shanghai Jiao Tong University.

### TUNEL assay

The mice tumor tissue samples were fixed in 10% formalin for 48 h, dehydrated by ethanol, and embedded in paraffin. In situ TUNEL staining of tissue sections was carried out using a TUNEL kit (Roche, GER) according to the manufacturer’s protocol and the apoptotic cells were those had brown-stained nuclei. The apoptotic index was calculated by the number of apoptotic cells/total number of nucleated cells × 100%.

### Chemicals

As mentioned in the reference^12^, CuET is synthesized directly from aqueous solutions of diethyldithiocarbamate sodium salt and copper (II) chloride. All chemical reagents used in this study are shown in Supplementary Table 2.

### Additional methods

Additional materials and methods are detailed in the supplemental materials, including cell culture and transfection (the sequences for RNA Oligos are shown in Supplementary Table 3.), cell viability assay, flow cytometry analysis, colony formation assay, dual-luciferase activity reporter assay, RNA extraction, and quantitative real-time PCR (primer sequences are listed in Supplementary Table 4.), high-throughput RNA-seq analysis, protein extraction, Western blot, immunohistochemistry, lactate, ATP and glucose uptake assays, seahorse metabolic analysis, co-immunoprecipitation (co-IP) and mass spectrometry, and ubiquitination assay and statistics.

## Results

### CuET inhibits cell viability and induces G2/M-phase arrest and apoptosis in CRC cells

**Fig. 1:**
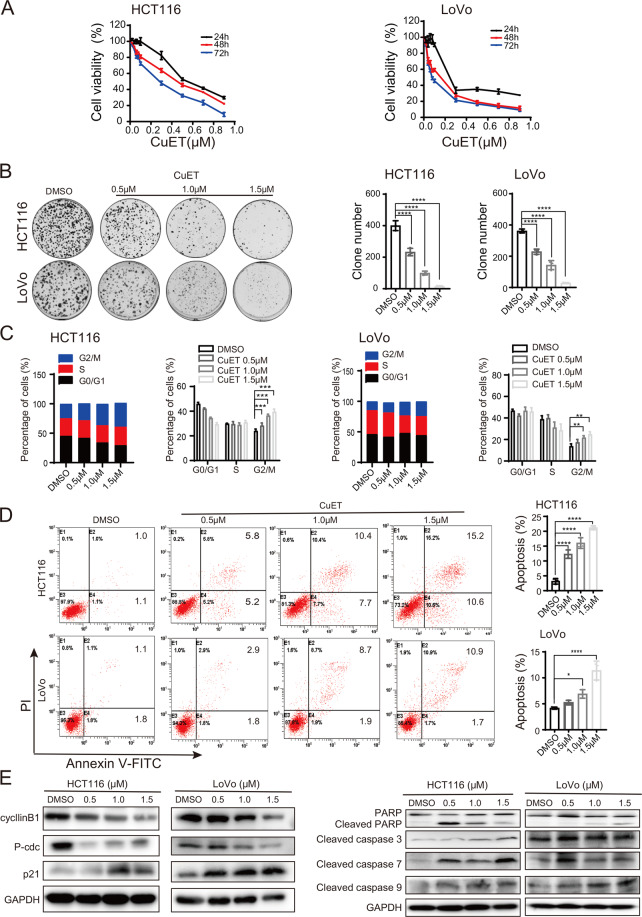
Effects of CuET on cell viability, cell cycle distribution, and apoptosis of CRC cell lines. **a** HCT116 and LoVo cells were incubated with different concentrations of CuET for 24 h, 48 h, or 72 h, and the cell viability was determined. **b** HCT116 and LoVo cells were treated with different concentrations of CuET (0.5 μM, 1.0 μM, 1.5 μM) or DMSO for 24 h, respectively; cells were then seeded as single cells and grown in drug-free medium for 14 days. Colony formation assay was carried out by crystal violet staining. The number of colonies was shown at right panels. **c**, **d** HCT116 and LoVo cells were treated with different concentrations of CuET; the cell cycle distribution (**c**) and apoptosis (**d**) were analyzed by flow cytometry. **e** Western blot of cyclin B1, P-cdc, P21, PARP, and cleaved PARP, cleaved caspase 3, caspase 7, and caspase 9 from HCT116 and LoVo cells treated with different concentrations of CuET and the controls. Data in **a**–**d** are shown as the mean ± SD from three independent experiments, *p < 0.05, **p < 0.01, ***p < 0.001, ****p < 0.001.

The cell viability of CRC was inhibited by CuET in a time- and dose-dependent manner (Fig. 1a). The IC50 values of CuET induced cytotoxicity in a group of CRC cell lines were lower than those of two normal colorectal epithelial cells, NCM460 and FHC, and a normal gastric epithelial cell, GES1, which indicated CuET might have a higher toxicity to CRC cells (Supplementary Fig. S1). In addition, the clone formation of CuET treated cells was significantly reduced in a dose-dependent manner compared with DMSO control group, indicating that the proliferation of CRC cells was dramatically modulated (Fig. 1b). These results show that the proliferation of CRC cells is effectively inhibited by CuET.

To investigate the mechanisms by which the phenotype of CRC cells was affected, flow cytometry was performed to assess cell cycle and apoptosis after treatment by CuET. As shown in Fig. 1c, the proportion of cell population was significantly increased in G2/M phase, and the apoptosis was markedly induced (Fig. 1d) in a dose-dependent manner in CuET treated group compared with the control. Notablely, Western blot showed the levels of cleaved PARP, caspase 3, caspase 7 and caspase 9 and p21 were markedly increased while cyclin B1 and P-cdc were greatly decreased upon CuET treatment (Fig. 1e). Finally, the results indicate that CuET treatment is able to lead to a profound G2/M arrest and apoptosis in CRC cells in vitro.

### CuET inhibits tumor growth in vivo

**Fig. 2:**
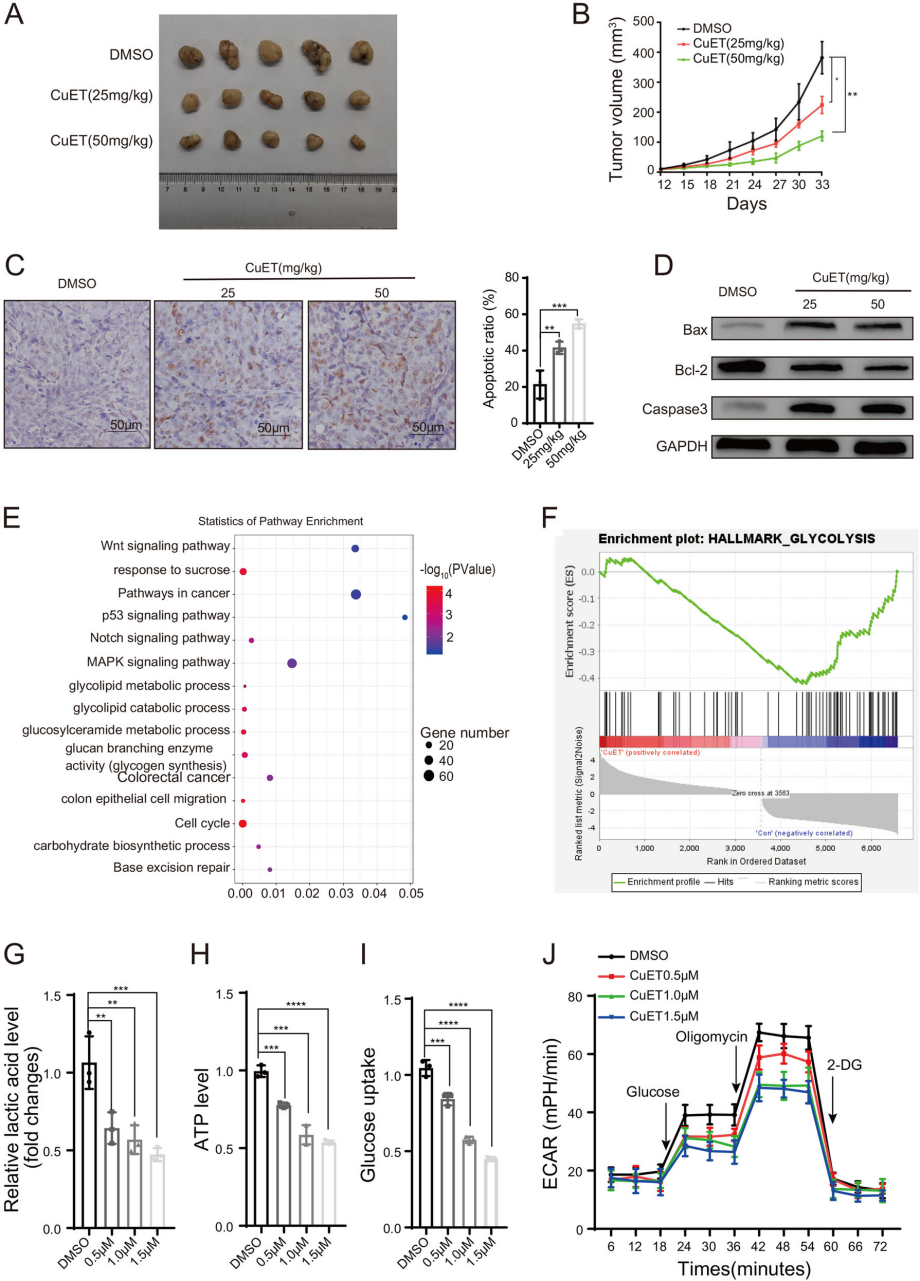
CuET suppresses tumor growth and induces apoptosis in vivo and attenuates Warburg effect in CRC cells. **a** Representative images of nude mice tumors in each group. **b** Tumor volume was measured from the 12th day after tumor cells were inoculated in each group (*n* = 5). **c**, **d** Apoptosis induction was examined by TUNEL assay and Western blot. TUNEL staining showed apoptosis in tumor tissue (the apoptotic cells were expressed in brown) (**c**). Western blot of Bax, Bcl-2, Caspase 3 from the lysates of the tumors in the primary tumor xenograft mice (**d**). **e** Bulb map of KEGG and GO pathway analysis for HCT116 cells treated with CuET, differential expression of genes related significantly enriched signaling pathway. X-axis represented the ratio of enriched differential genes in each pathway. Y-axis showed the name of statistics pathway enrichment. The area of each node represented the number of enriched differential genes. The p-value was indicated by different color changes from blue to red. **f** GSEA used to identify the differential gene profiles between HCT116 treated with CuET and controls. **g**–**i** Extracellular lactate production (**g**), cellular ATP level (**h**), and cellular glucose uptake (**i**) were measured in HCT116 cells treated with CuET. **j** Extracellular acid ratio (ECAR) upon cells was measured after HCT116 treated with different concentrations of CuET. Data in **b**, **c**, **g**–**j** are presented as the mean±SD from at least three independent experiments, **p < 0.01, ***p < 0.001, ****p < 0.0001.

To investigate the therapeutic potential of CuET in CRC, the CuET-treated CRC model and its control were developed. We found that the tumor volumes of CuET treated xenografts were significantly smaller compared with the DMSO control group (Fig. 2a). The quantification result is shown in Fig. 2b. Notably, 50 mg/kg CuET treatment group exhibited the highest percentage of apoptotic cells using TdT-mediated DUTP Nick-End Labeling (TUNEL) staining. In contrast, DMSO treatment group showed relatively few TUNEL positive cells (Fig. 2c). Furthermore, CuET was observed to promote Bax and caspase 3 protein level and suppress Bcl-2 expression (Fig. 2d). These findings suggest that CuET not only inhibits cell proliferation through regulation of the G2-M transition, but also induces apoptosis in CRC cells.

### CuET attenuates Warburg effect in CRC cells

To identify CuET downstream effectors, RNA sequencing (RNA-seq) was performed to assay the gene expression profiles using HCT116 treated with 1.0 μM CuET or DMSO as control for 24 h. A total of 6584 differentially expressed genes (*p* ≤ 0.05) were detected after CuET treatment in CRC cells. Pathway enrichment analysis showed the 15 most abundant enriched pathways including glycolipid metabolic and catabolic process (Fig. 2e). Gene set enrichment analysis (GSEA) indicated that the gene sets related to glycolysis negatively correlated with CuET treatment in CRC cells (Fig. 2f).

To validate the above findings, we treated HCT116 with different concentrations of CuET (0.5 μM, 1.0 μM, 1.5 μM) for 24 h. We found that CuET significantly reduced the lactate production, ATP level, and glucose consumption in HCT116 and LoVo cells compared with the control group (DMSO treatment) in a dose-dependent manner (Fig. 2g–i, Supplementary Fig. S2a–c). The real-time glycolytic rate in HCT116 and LoVo cells treated with CuET (0.5 μM, 1.0 μM, 1.5 μM) for 24 h was further examined using a Seahorse extracellular flux analyzer, which showed that CuET was able to markedly decrease the glycolytic rate (Fig. 2j, Supplementary Fig. S2d). However, CuET had no significant effect on oxygen consumption rate (OCR) in HCT116 and Lovo cells (Supplementary Fig. S2e, f). Collectively, these results demonstrate that CuET is capable of inhibiting CRC glycolysis.

### ALDH1A3 is a potential target gene of CuET

**Fig. 3:**
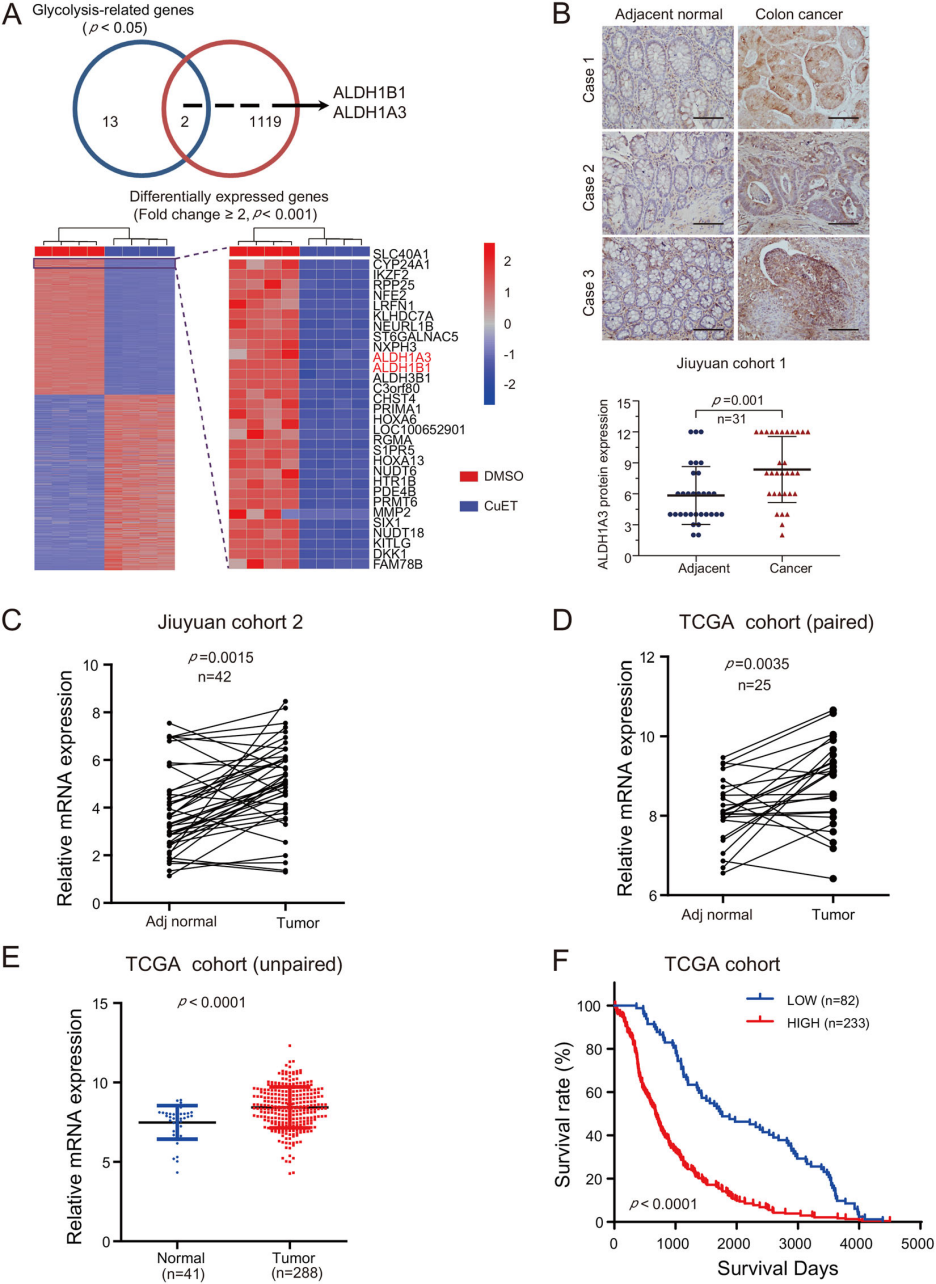
ALDH1A3 is the potential target gene of CuET and is correlated with prognosis. **a** Overlap of differentially expressed genes regulated by CuET and glycolysis enzymes. Heatmap revealing the differentially expressed genes in four replicates of the control- and CuET-24h treated HCT116 cells. Each row represents the relative level of expression of a single gene in all samples. The red and blue blocks represent up- and down-regulated genes, respectively, relative to the control cells. **b** Expression of ALDH1A3 in colorectal cancer tissues and paired normal tissues using IHC analysis. *n* = 31. Scale bars, 100 mm (top panel). ALDH1A3 protein expression was shown in the lower panel. **c** RT-qPCR showed that ALDH1A3 mRNA expression was upregulated in CRC compared to paired adjacent normal tissues in Jiuyuan cohort 2. **d**, e RNA-seq data from the TCGA study also showed that ALDH1A3 expression was up-regulated in CRC compared with adjacent normal tissues (paired (**d**) and unpaired (**e**) samples). **f** Prognostic value of ALDH1A3 expression was analyzed in CRC patients from TCGA study.

In order to further explore the molecular mechanisms of how CuET affects CRC, we analyzed 1119 differentially expressed genes after CuET treatment (Fold change ≥2, *p* < 0.001) and glycolysis-related genes (*p* < 0.05) regulated by CuET using KEGG_GLYCOLYSIS gene sets, and found that 2 genes were down-regulated (BioProject: PRJNA637929). As shown in Fig. 3a, we identified that ALDH1A3 and ALDH1B1 are potential candidates. Disulfiram (DSF) has been used clinically for more than 70 years in the treatment of alcoholism. DSF can irreversibly inhibit the liver aldehyde dehydrogenase, leading to accumulation of acetaldehyde after alcohol intake^28,29^. So far, 19 ALDH subtypes with different chromosome positions have been detected in human, including 1A1, 1A2, 1A3, 1B1, 1L1, 1L2, 2, 3A1, 3A2, 3B1, 3B2, 4A1, 5A1, 6A1, 7A1, 8A1, 9A1, 16A1, and 18A1. Recent studies have shown that ALDHs are related to tumor prognosis and are considered as a potential novel prognostic marker of cancer^30^. Based on our RNA-seq analyses, in addition to ALDH1A3 and ALDH1B1, we found that expressions of ALDH18A1, ALDH3B1, and ALDH5A1 decreased significantly after HCT116 cells were treated with CuET. Next, using RT-qPCR, we confirmed that expression of ALDH1A3 was also markedly reduced in other CRC cell lines (Supplementary Fig. S3a–d). We then examined ALDH1A3 expression in CRC cells and NCM460 cells, and found that the protein level of ALDH1A3 was higher in HCT116 and LoVo cells than in DLD1, RKO, SW480, and SW620 using Western blot (Supplementary Fig. S3e). To further validate the pathologic significance of ALDH1A3 in colorectal cancer, we detected and compared ALDH1A3 expression by immunohistochemical (IHC) staining assay in 31 paired paraffin-embedded colorectal cancer and adjacent tissues. ALDH1A3 expression was higher in colorectal cancer tissues than that in adjacent tissues in Shanghai Ninth People’s Hospital cohort 1 (Jiuyuan cohort 1) (Fig. 3b). RT-qPCR showed that ALDH1A3 mRNA expression was significantly upregulated in primary CRC tumors. Moreover, the upregulation of ALDH1A3 mRNA in CRC was verified using Jiuyuan cohort 2 (*n* = 42; *p* = 0.0015) (Fig. 3c) and the TCGA cohort (*n* = 25; *p* = 0.0035) (Fig. 3d). Meanwhile, TCGA studies also showed a significant upregulation of ALDH1A3 mRNA in CRC (*n* = 288) compared with unpaired adjacent normal tissues (*n* = 41) (Fig. 3e). Finally, Kaplan-Meier survival curves showed that CRC patients with high ALDH1A3 expression had poorer survival than those with low expression (*p* < 0.0001; Fig. 3f). In summary, these findings suggest that ALDH1A3 modulated by CuET may be associated with progression of CRC.

### ALDH1A3 alters the phenotypes of CRC cells

**Fig. 4:**
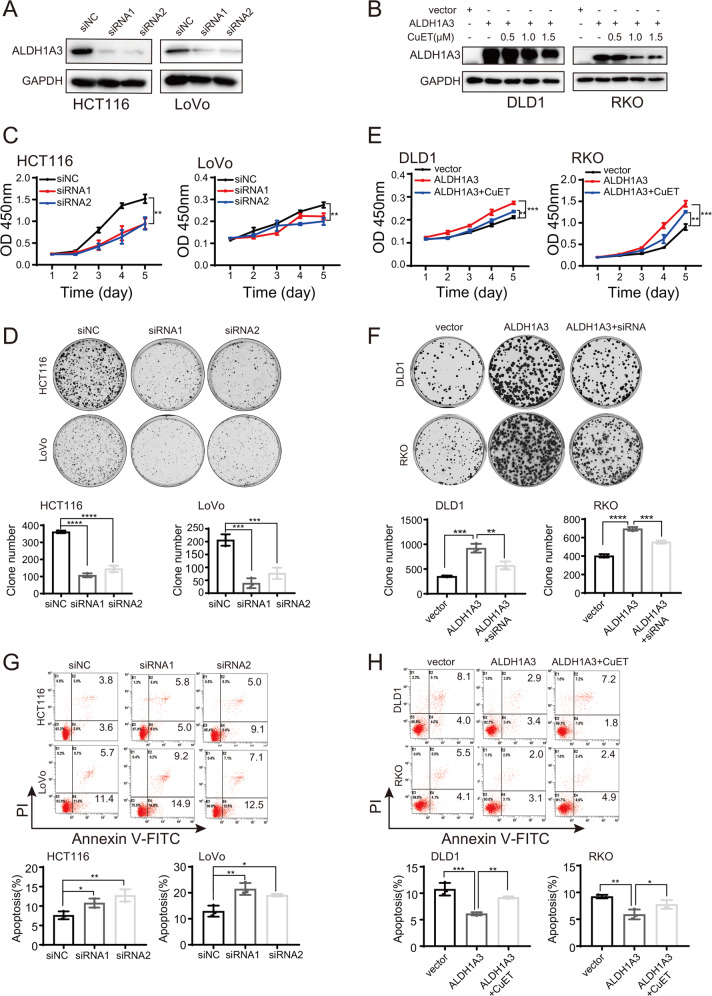
ALDH1A3 alters the phenotypes of CRC cells. **a** Downregulation of ALDH1A3 by transfection of siRNAs was confirmed by Western blot in HCT116 and LoVo cells. **b** Upregulation of ALDH1A3 by transfection of ALDH1A3 plasmids was confirmed by Western blot in DLD1 and RKO cells. **c**, **d** Knockdown of ALDH1A3 significantly inhibited proliferation of HCT116 and LoVo by CCK8 **(c)** and colony formation assay (**d**). **e**, **f** Overexpression of ALDH1A3 significantly increased proliferation of DLD1 and RKO by CCK8 (**e**) and colony formation assay (**f**), and could be partially counteracted by CuET treatment and ALDH1A3 knockdown, respectively. **g**, **h** Flow cytometry showed that knockdown of ALDH1A3 promoted cell apoptosis (**g**), while overexpression of ALDH1A3 inhibited apoptosis and was partially counteracted by 1.0 μM CuET treatment (**h**). Data in **c**–**h** are presented as the mean ± SD of three independent experiments, *p < 0.05, **p < 0.01, ***p < 0.001, ****p < 0.0001.

To further study the role of ALDH1A3 in CRC, HCT116 and LoVo cells were transfected with ALDH1A3 siRNAs or their expressing plasmids to explore its gain and loss of functions in vitro, as shown in Fig. 4a, b. RNAi-mediated knockdown of ALDH1A3 in those cells significantly inhibited cell viability and clonogenicity (Fig. 4c, d), whereas restoring ALDH1A3 expression in DLD1 and RKO cells markedly enhanced the effects, and cell viability was partially counteracted by CuET treatment (Fig. 4e). Additionally, the promotion of cell clonogenicity in the DLD1 and RKO cells with ALDH1A3 overexpression were partially counteracted by ALDH1A3 knockdown (Fig. 4f). Moreover, the similarly affected tendency in apoptosis was also observed in these CRC cells (Fig. 4g, h). These results suggest that proliferation induced by ALDH1A3 is associated with its role in CRC cells.

### CuET inhibits ALDH1A3 by selectively enhancing expressions of miR-16-5p and miR-15b-5p

Based on the above findings, we next explore the mechanism by which CuET modulates ALDH1A3. Luciferase reporter plasmid GV238-ALDH1A3 was constructed, which contains the promoter region of ALDH1A3. Luciferase assay showed that CuET treatment had no effect on transcriptional activity of GV238-ALDH1A3 in CRC cells compared with the control (Supplementary Fig. S4a), suggesting that inhibition of ALDH1A3 induced by CuET does not occur at the transcriptional level.

**Fig. 5:**
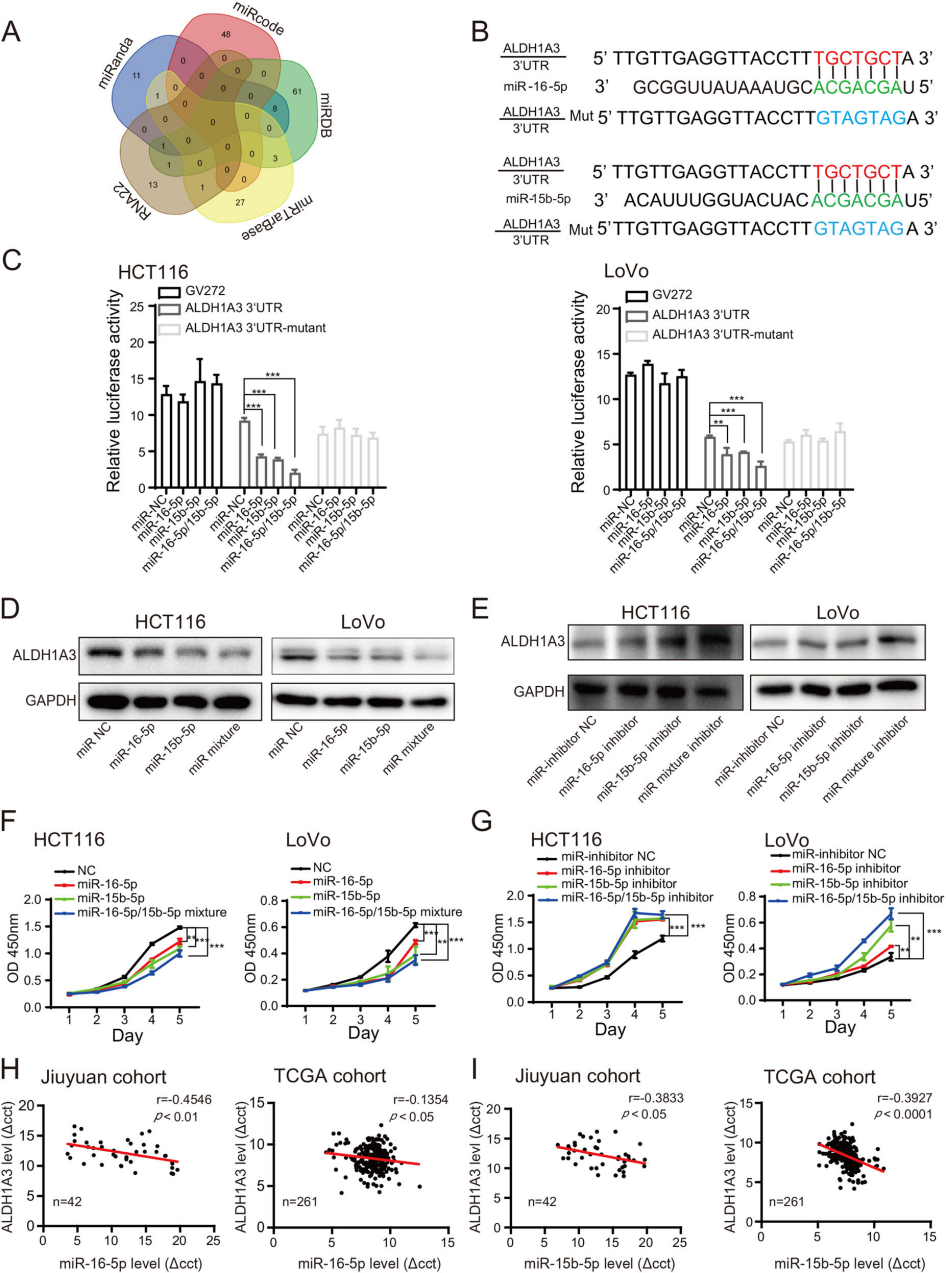
CuET inhibits ALDH1A3 by selectively enhancing expressions of miR-16-5p and miR-15b-5p. **a** Venn diagram showed data for each database intersection. **b** The potential binding site for miR-16-5p (up) and miR-15b-5p (down) and the mutated sequence in the ALDH1A3 3′UTR for the seed region were shown in blue. **c** Luciferase activity was measured in HCT116 and LoVo cells transfected with miR-16-5p, miR-15b-5p, miR-16-5p/miR-15b-5p mixture mimics or control miRNA. The luciferase reporter plasmid expressing wild-type, mutant human ALDH1A3 3′UTRs and empty vector (GV272) were used. The luciferase activity was normalized to the control miRNA. **d** Western blot of ALDH1A3 from HCT116 and LoVo cells transfected with miR-16-5p, miR-15b-5p, miR-16-5p/miR-15b-5p mixture mimics or control miRNA. **e** Western blot of ALDH1A3 from HCT116 and LoVo cells transfected with miR-16-5p, miR-15b-5p, miR-16-5p/miR-15b-5p mixture inhibitor or control miRNA. **f** Cell proliferation assay was performed in HCT116 and LoVo cells transfected with miR-16-5p, miR-15b-5p, miR-16-5p/miR-15b-5p mixture mimics or control miRNA. **g** Cell proliferation assay was performed in HCT116 and LoVo cells transfected with miR-16-5p, miR-15b-5p, miR-16-5p/miR-15b-5p mixture inhibitor or control miRNA. **h**, **i** Levels of miRNA-16-5p (**h**) and miRNA-15b-5p (**i**) were negatively correlated with the mRNA expression of ALDH1A3 in Jiuyuan cohort and TCGA cohort using linear regression analysis. Data are presented as the mean±SD of three independent experiments, **p < 0.01, ***p < 0.001.

It has been reported that microRNAs (miRNAs) are short non-coding RNA that often regulate gene expression by binding to the RISC complex and directing sequence-specific cleavage of target mRNA or repressing the target mRNA translation^31^. Thus, we hypothesized that CuET-induced inhibition of ALDH1A3 expression might be caused by dysregulated miRNAs. To test this hypothesis, we employed six miRNA prediction databases, including miRanda (http://www.microrna.org/microrna/home.do), miRcode (http://www.mircode.org/index.php), miRDB (http://www.mirdb.org/index.html), RNA22 (https://cm.jefferson.edu/rna22-full-sets-of-predictions), miRTarBase (http://mirtarbase.mbc.nctu.edu.tw/php/index.php),Targetscan (http://www.targetscan.org/vert_72) to predict putative binding of miRNAs to the 3’UTR of ALDH1A3 transcript. Fifteen miRNAs were predicted in greater than or equal to three databases overlap (Fig. 5a, Supplementary Table 5), all of which were therefore validated using real-time PCR. Among fifteen miRNAs, we found that six miRNAs in HCT116 and LoVo cells were greatly downregulated compared with those in NCM460 cells (Supplementary Fig. S4b). Notably, we found that expressions of miR-16-5p and miR-15b-5p were the most significantly upregulated in response to CuET intervention in these CRC cells compared with the respective controls (Supplementary Fig. S4c). Target prediction programs and sorting algorithm suggest that both miR-16-5p and miR-15b-5p are the potential specific targets in the seed regions within the 3′UTR regions of ALDH1A3 genes. Moreover, we found that the 3′UTR of ALDH1A3 harbors the same binding sites for these two miRNAs (Fig. 5b). Luciferase reporter assay demonstrated that miR-16-5p or miR-15b-5p suppressed the luciferase activity in HCT116 and LoVo cells transfected with wild-type ALDH1A3 reporter plasmids, but not with the mutant ones. Meanwhile, we found the mixture of miR-16-5p/15b-5p inhibited the luciferase activity of wild-type ALDH1A3 reporter plasmids most significantly (Fig. 5c). These findings suggest that CuET-induced ALDH1A3 inhibition is closely responsible for the enhanced expressions of miR-16-5p and miR-15b-5p.

To test this hypothesis, equal amounts of miR-16-5p (100 pmol), miR-15b-5p (100 pmol), or miR-16-5p/15b-5p (50 pmol each) mixture mimics were transfected into HCT116 and LoVo cells as shown in Supplementary Fig. S4d, e. We found that expressions of ALDH1A3 at mRNA and protein levels were markedly reduced in these CRC cells after transfection with miR-16-5p, miR-15b-5p mimic, or both mimics compared with the respective controls (Supplementary Fig. S4f, Fig. 5d). These results suggest that the inhibitory effect of miR-16-5p/15b-5p on ALDH1A3 was synergistic. However, the mRNA and protein expressions of ALDH1A3 were significantly increased in HCT116 and LoVo cells transfected with miR-16-5p inhibitor (100 pmol) or miR-15b-5p inhibitor (100 pmol), and a synergistic effect was observed when cells were cotransfected with miR-16-5p/15b-5p mixture inhibitors (50 pmol each) (Supplementary Fig. S4g–i, Fig. 5e). These results confirm the synergistic effects of miR-16-5p and miR-15b-5p on the expression of ALDH1A3 in CRC cells.

To investigate the role of miR-16-5p and miR-15b-5p in CRC, HCT116, and LoVo cells were transfected with miR-16-5p and miR-15b-5p mimics or inhibitors, and their effects on the cell proliferation were analyzed. Cell viability assay showed that cell proliferation was significantly decreased in HCT116 and LoVo cells transfected with miR-16-5p or 15b-5p mimic (Fig. 5f); in contrast, the effects were markedly increased in these cells transfected with miR-16-5p or 15b-5p inhibitor (Fig. 5g). Notably, these CRC cells transfected with mixture mimics or mixture inhibitors conferred the strongest effect compared with the above individually, indicating that miR-16-5p and miR-15b-5p exert synergistic effects on cell proliferation in CRC cells.

Finally, using Jiuyuan cohort and TCGA cohort, we next analyzed the correlation between miRNAs and ALDH1A3 in CRC patients. As shown in Fig. 5h, i, we found ALDH1A3 mRNA expression was negatively associated with miR-16-5p or miR-15b-5p expression using linear regression analysis. Taken together, these findings suggest that the increased expressions of miR-16-5p and miR-15b-5p may function as suppressors upon CuET treatment, which negatively regulate ALDH1A3 in CRC.

### ALDH1A3 directly interacts with PKM2 and inhibits its ubiquitination

**Fig. 6:**
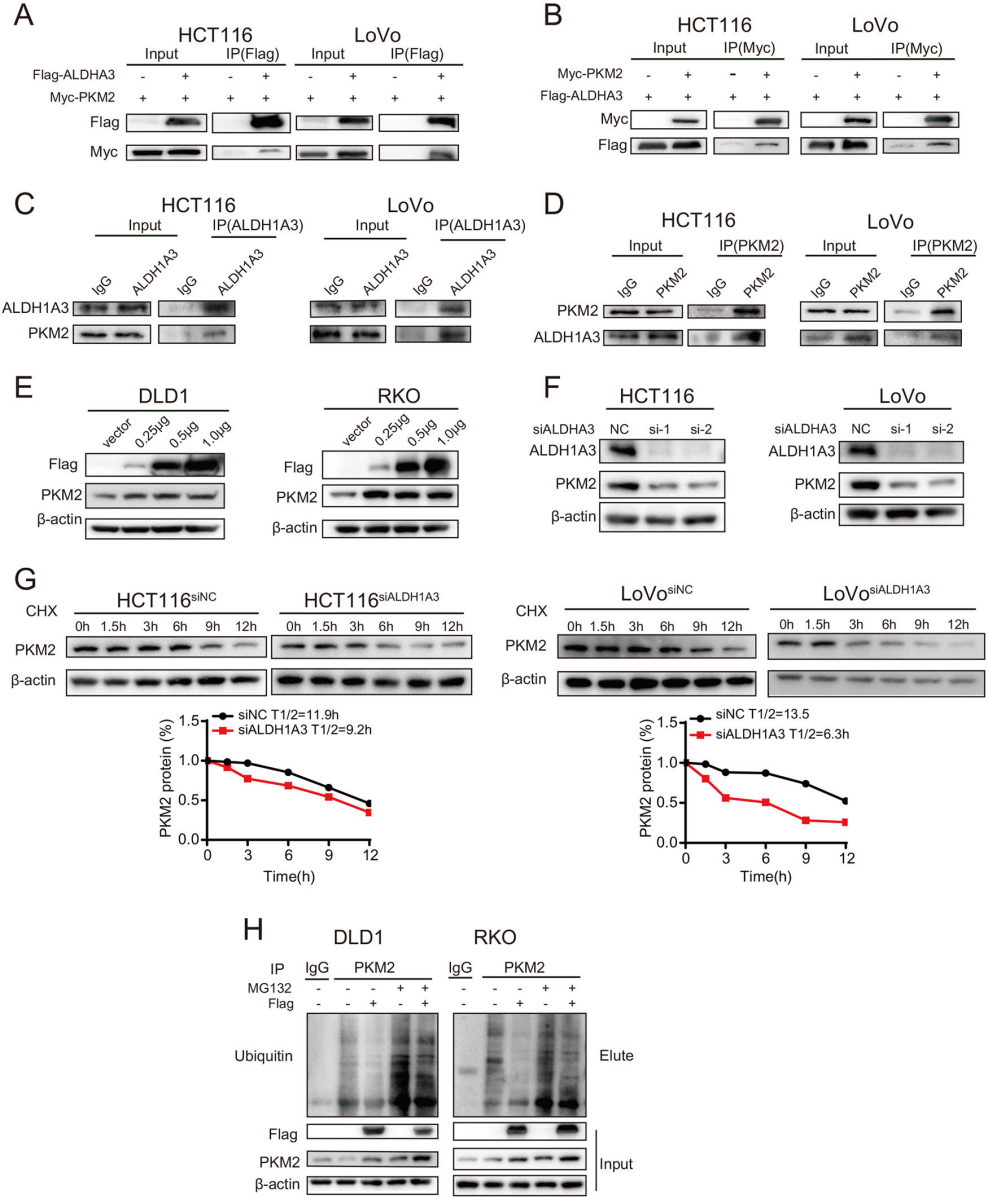
ALDH1A3 directly interacts with PKM2 and inhibits its ubiquitination. **a**, **b** HCT116 and LoVo cells were co-transfected with Flag-tagged ALDH1A3 and Myc-tagged PKM2 and subjected to IP of Flag (**a**) or Myc (**b**). **c**, **d** Reciprocal co-IP of ALDH1A3 (**c**) and PKM2 (**d**) showing the physical interaction between endogenously expressed ALDH1A3 and PKM2 in HCT116 and LoVo cells. **e** Ectopic expression of ALDH1A3 increased the PKM2 protein levels in DLD1 and RKO cells. **f** After siALDH1A3 was transfected, PKM2 protein expression was decreased in HCT116 and LoVo cells. **g** HCT116 and LoVo cells were transfected with siALDH1A3 or its control, then incubated with 100 mg/ml Cycloheximide (CHX). The levels of PKM2 and β-actin were detected by immunoblotting at the indicated time (up panel), and quantification of PKM2/β-actin ratio is shown (down panel)**. h** ALDH1A3 increased the PKM2 level by inhibiting its ubiquitin-mediated degradation.

Since CuET can both regulate glycolysis and reduce the expression of ALDH1A3, we next hypothesized that ALDH1A3 was responsible for the glycolysis. To test this possibility, we analyzed the mRNA levels using RT-qPCR of 5 candidate genes related to glycolysis in DLD1 and RKO cells transfected with ALDH1A3 plasmid or HCT116 and LoVo cells transfected with siALDH1A3. Notably, altered expressions of ALDH1A3 did not show a marked effect on the expression of these genes including ALDOA, ENO1, LDHA, PDK1, and PKM2 (Supplementary Fig. S5a, b). These results suggest that ALDH1A3 may modulate glycolysis at post-transcriptional level. In order to assay the direct interacting partners of ALDH1A3, we conducted co-IP with antibody direct against endogenous ALDH1A3 to pull down potential interacting proteins from HCT116 cells, coupled with LC-MS/MS analysis. Among the identified candidates, 3 glycolysis associated genes including ALDOA, PKM2, and ENO1 may interact with ALDH1A3 (Supplementary Fig. S5c–e, Supplementary Table 6). To validate this interaction, CRC cells were transfected with Flag-ALDH1A3 or Myc-PKM2 or co-transfected with both plasmids and then a set of co-IP assays were performed. The pull-down was performed with anti-Flag antibody in HCT116 and LoVo cells over-expressing Flag-tagged ALDH1A3 and the physical association of ALDH1A3 and PKM2 was confirmed in these cells (Fig. 6a). Next, the reverse co-IP assays using the anti-Myc antibody confirmed the interaction of PKM2 with ALDH1A3 (Fig. 6b). Co-IP assays were further performed using endogenous proteins and it was found that both PKM2 and ALDH1A3 could be pulled down with either anti-PKM2 antibody or anti-ALDH1A3 antibody (Fig. 6c, d). These results indicate that PKM2 is able to physically interact with ALDH1A3 in vivo.

In light of these findings, we next tested whether ALDH1A3 could alter PKM2 expression. Given that ubiquitin-proteasome system is the main pathway of protein degradation in cells, we hypothesized that ALDH1A3 may regulate PKM2 via the ubiquitin-proteasome system. We also observed that PKM2 protein levels in ALDH1A3-transfected DLD1 and RKO cells were greatly increased in a dose-dependent manner (Fig. 6e), whereas knockdown of ALDH1A3 expression in HCT116 and LoVo cells markedly decreased PKM2 protein levels (Fig. 6f), indicating that ALDH1A3 positively regulated PKM2 at posttranscriptional level.

Then, cycloheximide (CHX) was added into HCT116 and LoVo cells to inhibit protein synthesis to observe whether effects of PKM2 expression were affected by ALDH1A3. We found that the half-life of endogenous PKM2 protein was significantly shorter than that in the control group in HCT116 and LoVo cells after transfection of ALDH1A3 siRNA (9.2 h, 6.3 h, respectively) (Fig. 6g). Finally, we performed ubiquitination assays to investigate whether the level of PKM2 was increased by the ALDH1A3-PKM2 interaction. The ubiquitination and protein level of PKM2 were examined in the presence or absence of ALDH1A3 and/or the proteasome inhibitor MG132. We found that ALDH1A3 expression was able to decrease the ubiquitination level of PKM2 in DLD1 and RKO cells using anti-PKM2 IP lysates; however, ALDH1A3 expression coupled with MG132 treatment increased the level of PKM2 compared with the respective control (Fig. 6h). Taken together, these results indicate that ALDH1A3-PKM2 interaction stabilizes PKM2 at the protein level by inhibiting its ubiquitin-mediated degradation.

### miR-16-5p, miR-15b-5p/ALDH1A3 axis regulates glycolysis in vitro

**Fig. 7:**
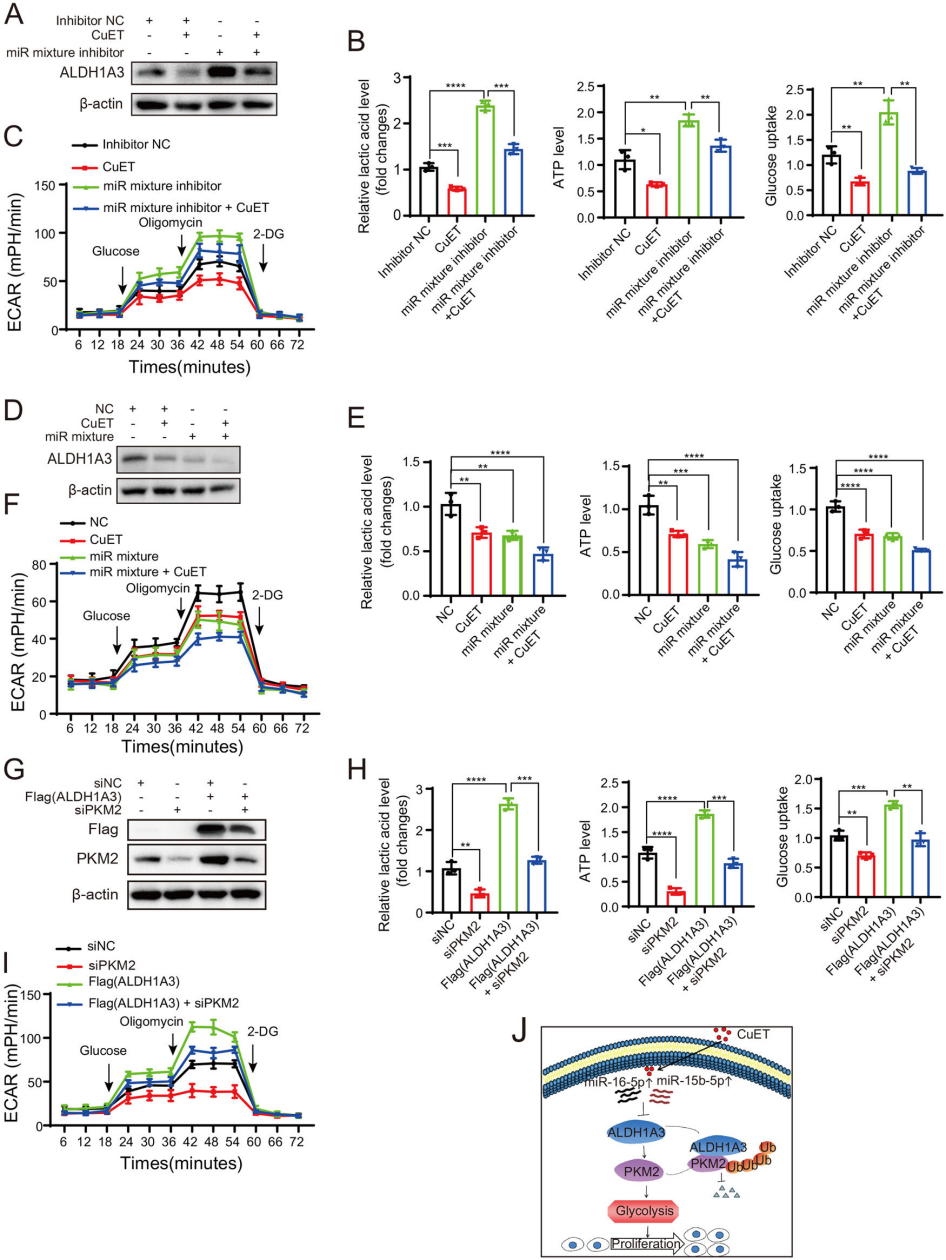
miR-16-5p, miR-15b-5p/ALDH1A3 axis regulates glycolysis in vitro. **a** Western blot showed the expression of ALDH1A3 in HCT116 cells treated with CuET, transfected with inhibitor NC, mixture inhibitors, or mixture inhibitors plus CuET treatment. **b**, **c** Their lactate production, ATP level, glucose uptake (**b**) and ECAR (**c**) were determined respectively. **d** Western blot showed the expression of ALDH1A3 in HCT116 cells treated with CuET, transfected with NC, mixture mimics or mixture mimics plus CuET treatment. **e**, **f** Their lactate production, ATP level, glucose uptake (**e**) and ECAR (**f**) were determined respectively. **g** Western blot showed the expression of PKM2 in HCT116 cells transfected with siNC, PKM2 siRNA, Flag-tagged ALDH1A3 plasmid, or PKM2 siRNA plus ALDH1A3 plasmid. **h**, **i** Their lactate production, ATP level, glucose uptake (**h**), and ECAR (**i**) were determined respectively. Data shown are mean±SD of three independent experiments, **p < 0.01, ***p < 0.001, ****p < 0.0001. **j** The working model depicts the mechanism of CuET modulating glycolytic metabolism through miR-16-5p and miR-15b-5p/ALDH1A3/PKM2 axis.

Aerobic glycolysis, also known as Warburg effect, is a common feature of glucose metabolism in cancer cells. The glycolysis of tumor cells under aerobic conditions, instead of tricarboxylic acid cycle, produces a large amount of lactic acid and increases biosynthesis^32^. Based on the expression of glycolysis gene PKM2 regulated by miR-16-5p, miR-15b-5p/ALDH1A3 axis, we tested whether this axis modulates the glycolytic phenotype in CRC cells. As shown in Fig. 7a and Supplementary Fig. S6a, miR-16-5p/15b-5p mixture inhibitors markedly increased ALDH1A3 expression in HCT116 and LoVo cells using Western blot assays, which could be partially reversed by CuET treatment. Meanwhile, lactate production, ATP level, glucose uptake (Fig. 7b, Supplementary Fig. S6b), and extracellular acidification rate (ECAR) that reflects the extracellular acid production capacity of these cells were greatly increased after treatment with the mixture inhibitors compared with the respective controls (Fig. 7c, Supplementary Fig. S6c). These effects could be partially reversed by CuET treatment. In contrast, the mixture mimics reduced ALDH1A3 expression in HCT116 and LoVo cells demonstrated by Western blot assays, while CuET treatment showed a synergistic role in this effect (Fig. 7d, Supplementary Fig. S6d). By transfection of the mixture mimics, lactate production, ATP level, glucose uptake, and ECAR (Fig. 7e, Supplementary Fig. S6e, and Fig. 7f, Supplementary Fig. S6d) were decreased. Moreover, all of these effects were greatly enhanced by CuET treatment. Notably, using rescued assays, we found that knockdown of PKM2 expression was partially abolished by ectopic expression of ALDH1A3 (Fig. 7g, Supplementary Fig. S6g), followed by an increase in the lactate production, ATP level, glucose uptake, and ECAR to varying extents (Fig. 7h, Supplementary Fig. S6h and Fig. 7i, Supplementary Fig. S6i). Taken together, our results indicated that the miR-16-5p, miR-15b-5p/ALDH1A3 axis regulate glycolysis through PKM2 in CRC cells.

## Discussion

Current treatments of CRC include surgery, radiotherapy, and chemotherapy to prevent its spread and growth, and chemotherapy is still a major choice in clinic, especially for the unresectable stage and metastatic CRC^33^, but the development of drug resistance remains the greatest limitation in chemotherapy^34^. The research and development of anti-tumor drugs has the problems of long cycle and large investment; therefore, it is highly worthy to repurpose traditional drugs in treatment of tumor. Currently, multiple studies show that non-tumor drugs familiar to clinicians may also have active anti-tumor effects. For example, metformin for diabetes is effective for pancreatic cancer, lung cancer, and ovarian cancer^35–37^; aspirin for cardiovascular diseases is effective for hepatocellular carcinoma, prostate cancer, and gastric cancer etc^38–40^. Notably, we recently found that dichloroacetate (DCA), which was originally used to treat lactic acidosis, hereditary mitochondrial metabolism disorder and diabetes, may restore chemosensitivity in CRC cells^41–43^. Therefore, it is valuable to explore the pharmacological effects of existing drugs for saving medical resources, improving the life span and quality of life of patients by repurposing traditional drugs.

DSF, an antialcoholism drug that has been used for over 70 years, is found to have anti-tumor effect on a variety of tumors. The mechanism that DSF exerts its anti-alcoholic effect is that it forms disulfide bond with the active site of ALDH, which causes irreversible inhibition of ALDH^29^. However, although many targets and pathways have been proposed, such as NPL4^12^, ALDH^44^, Akt^10,11^, ROS^45,46^, JNK-p38^47^, NF-κB^8,48^, the anti-tumor mechanism of DSF in CRC is still far from completely elucidated. In vivo, oral disulfiram is mainly absorbed in the gastrointestinal tract and metabolized into diethyldithiocarbamate (DTC), S-methyl-N, and N-diethyldithiocarbamate (DETC). It is generally believed that the anti-cancer mechanism of DSF is to inhibit ALDH enzyme. However, the current research showed that intermediate metabolite, DTC-copper complex (CuET), plays an active anti-cancer role in vivo, without inhibition of ALDH activity^13^.

In this study, using GSEA analyses, we demonstrated that glycolytic pathways in CRC were significantly suppressed in response to CuET treatment. In combination with genomic, biochemical, and cellular biological analyses, it was proved that ALDH1A3 was the direct target gene of CuET treatment in CRC. The IC50 values of CuET were detected in a panel of CRC cell lines. CuET had similar or lower IC50 values in DLD1 and RKO cells with low ALDH1A3 expression compared with HCT116 and LoVo cells with high ALDH1A3 expression. We speculated that the effect of CuET in CRC cells may be independent of ALDH1A3. CuET treatment or knockdown of ALDH1A3 in CRC cells markedly inhibited cell growth while promoting cell apoptosis, which was further confirmed using xenograft mouse models. The data showed that CuET had active anticancer effect in CRC, and ALDH1A3 might be a driving oncogene in CRC development for targeting.

Notably, we analyzed the mechanisms by which CuET mediates the ALDH1A3 pathway suppression. CuET does not affect the transcription of ALDH1A3. It has been reported that miR-7 modulates ALDH1A3 level in breast cancer^49^. Using bioinformatics and functional studies, we elucidated that both miR-16-5p and miR-15b-5p targeted ALDH1A3 selectively increased due to CuET treatment, hence could modulate cancer cells growth. Thus, CuET plays an anti-cancer role via selectively targeting specific miRNAs and ALDH1A3 signaling pathway.

Tumor energy metabolism is characterized by the use of glycolysis, which consumes a large amount of glucose to produce high lactate, rather than the use of more efficient mitochondrial aerobic metabolism^50^. Pyruvate kinase (PK) catalyzes the conversion of phosphoenolpyruvate to pyruvate and yields ATP, which is the final rate-limiting step of glycolysis^51^. There are four isoforms of PK in mammalian cells: PKM1, PKM2, PKL, and PKR. The abnormal high expression of PKM2 is closely related to the occurrence and development of tumor. In addition to its function as a glycolytic enzyme, PKM2 is also involved in more cellular processes by interaction with other proteins in cytoplasm^52^. For example, PKM2 interacts with TRIM35 and fibroblast growth factor receptor 1 (FGFR1), thus changing cell metabolism and regulating signal transduction^53,54^. Furthermore, PKM2, as an important transcription factor, promotes the expression of oncogenes such as NF-κB, β-catenin, c-MYC and phosphorylates ERK1/2 in nuclei^55–57^.

Here, to further determine the mechanisms by which miRNAs/ALDH1A3 mediated CuET regulating glycolysis, we first performed qRT-PCR to confirm whether ALDH1A3 could promote the expression of glycolysis related genes including ALDOA, ENO1, PKM2, LDHA, PDK1. However, ALDH1A3 expression did not affect the mRNA levels of these genes, which indicated that ALDH1A3 regulated glycolysis related genes through a post-transcriptional mechanism. Using mass spectrometry coupled with co-IP data, we demonstrated that ALDH1A3 directly interacted with and stabilized PKM2 protein, thereby activating glycolytic metabolism.

In short, as shown in Fig. 7j, our results indicate that CuET treatment is capable of modulating the interaction between ALDH1A3 and PKM2 via miR-16-5p/15b-5p. Targeting this pathway may provide an effective strategy for the treatment of CRC. Biotransformation of CuET is often impacted by age, gender, liver disease, drugs, and others. Further clinical trials are warranted to demonstrate the concentrations of CuET applied in the current study are efficacious and tolerable as disulfiram in the management of CRC.

## Supplementary information

Supplementary materials and methods

Supplementary Table1-6

Supplementary Figure 1

Supplementary Figure 2

Supplementary Figure 3

Supplementary Figure 4

Supplementary Figure 5

Supplementary Figure 6

Supplementary figure legends

## References

[1] Wolf AMD (2018). Colorectal cancer screening for average-risk adults: 2018 guideline update from the American Cancer Society. CA Cancer J. Clin..

[2] Global Burden of Disease Cancer, C et al. Global, regional, and National Cancer incidence, mortality, years of life lost, years lived with disability, and disability-adjusted life-years for 29 Cancer Groups, 1990 to 2017: a systematic analysis for the global burden of disease study. *Jama Oncol*. **5**,1749–1768 (2019). 10.1001/jamaoncol.2019.2996 PMC6777271 31560378

[3] Petrakis IL (2006). Naltrexone and disulfiram in patients with alcohol dependence and comorbid post-traumatic stress disorder. Biol. Psychiatry.

[4] Wang C (2017). Disulfiram-loaded porous PLGA microparticle for inhibiting the proliferation and migration of non-small-cell lung cancer. Int. J. Nanomed..

[5] Najlah M (2017). Development and characterisation of disulfiram-loaded PLGA nanoparticles for the treatment of non-small cell lung cancer. Eur. J. Pharm. Biopharm..

[6] Wang Z (2017). Poly lactic-co-glycolic acid controlled delivery of disulfiram to target liver cancer stem-like cells. Nanomedicine.

[7] Hoda M, Pajaniradje S, Shakya G, Mohankumar K, Rajagopalan R (2016). Anti-proliferative and apoptosis-triggering potential of disulfiram and disulfiram-loaded polysorbate 80-stabilized PLGA nanoparticles on hepatocellular carcinoma Hep3B cell line. Nanomedicine.

[8] Li Y (2018). Disulfiram combined with copper inhibits metastasis and epithelial-mesenchymal transition in hepatocellular carcinoma through the NF-kappaB and TGF-beta pathways. J. Cell Mo. l Med..

[9] Liu P (2013). Disulfiram targets cancer stem-like cells and reverses resistance and cross-resistance in acquired paclitaxel-resistant triple-negative breast cancer cells. Br. J. Cancer.

[10] Zhang H (2010). Disulfiram treatment facilitates phosphoinositide 3-kinase inhibition in human breast cancer cells in vitro and in vivo. Cancer Res..

[11] Kim JY (2016). Disulfiram targets cancer stem-like properties and the HER2/Akt signaling pathway in HER2-positive breast cancer. Cancer Lett..

[12] Skrott Z (2017). Alcohol-abuse drug disulfiram targets cancer via p97 segregase adaptor NPL4. Nature.

[13] Skrott Z (2019). Disulfiram’s anti-cancer activity reflects targeting NPL4, not inhibition of aldehyde dehydrogenase. Oncogene.

[14] Sanderson SM, Locasale JW (2018). Revisiting the Warburg effect: some tumors hold their breath. Cell Metab..

[15] Husain SR, Han J, Au P, Shannon K, Puri RK (2015). Gene therapy for cancer: regulatory considerations for approval. Cancer Gene Ther..

[16] Okugawa Y, Grady WM, Goel A (2015). Epigenetic alterations in colorectal cancer: emerging biomarkers. Gastroenterology.

[17] Chen M (2019). CRISPR-Cas9 for cancer therapy: opportunities and challenges. Cancer Lett..

[18] Abbaszadeh Z, Cesmeli S, Biray Avci C (2020). Crucial players in glycolysis: cancer progress. Gene.

[19] Lu Z, Hunter T (2018). MetabolIc Kinases Moonlighting As Protein Kinases. Trends Biochem. Sci..

[20] Li X (2016). Mitochondria-translocated PGK1 functions as a protein kinase to coordinate glycolysis and the TCA cycle in tumorigenesis. Mol. Cell.

[21] Wang Y (2019). LncRNA LINRIS stabilizes IGF2BP2 and promotes the aerobic glycolysis in colorectal cancer. Mol. Cancer.

[22] Deng F (2019). Tumor-secreted dickkopf2 accelerates aerobic glycolysis and promotes angiogenesis in colorectal cancer. Theranostics.

[23] Marcato P (2011). Aldehyde dehydrogenase activity of breast cancer stem cells is primarily due to isoform ALDH1A3 and its expression is predictive of metastasis. Stem Cells.

[24] Schmidtova S (2019). Disulfiram overcomes cisplatin resistance in human embryonal carcinoma cells. Cancers (Basel).

[25] Marcato P (2015). Aldehyde dehydrogenase 1A3 influences breast cancer progression via differential retinoic acid signaling. Mol. Oncol..

[26] Cheng P (2016). FOXD1-ALDH1A3 signaling is a determinant for the self-renewal and tumorigenicity of mesenchymal glioma stem cells. Cancer Res..

[27] Feng H, Liu Y, Bian X, Zhou F, Liu Y (2018). ALDH1A3 affects colon cancer in vitro proliferation and invasion depending on CXCR4 status. Br. J. Cancer.

[28] Liu P (2013). Reply: cytotoxic effect of disulfiram/copper on human glioblastoma cell lines and ALDH-positive cancer-stem-like cells. Br. J. Cancer.

[29] Malka F, Dairou J, Ragunathan N, Dupret JM, Rodrigues-Lima F (2009). Mechanisms and kinetics of human arylamine N-acetyltransferase 1 inhibition by disulfiram. Febs J..

[30] Xu X (2015). Aldehyde dehydrogenases and cancer stem cells. Cancer Lett..

[31] Treiber T, Treiber N, Meister G (2019). Regulation of microRNA biogenesis and its crosstalk with other cellular pathways. Nat. Rev. Mol. Cell Biol..

[32] Koppenol WH, Bounds PL, Dang CV (2011). Otto Warburg’s contributions to current concepts of cancer metabolism. Nat. Rev. Cancer.

[33] Jamal R (2017). Peripheral and local predictive immune signatures identified in a phase II trial of ipilimumab with carboplatin/paclitaxel in unresectable stage III or stage IV melanoma. J. Immunother. Cancer.

[34] Galletti G, Leach BI, Lam L, Tagawa ST (2017). Mechanisms of resistance to systemic therapy in metastatic castration-resistant prostate cancer. Cancer Treat. Rev..

[35] Han H (2020). Metformin-induced stromal depletion to enhance the penetration of gemcitabine-loaded magnetic nanoparticles for pancreatic cancer targeted therapy. J. Am. Chem. Soc..

[36] Lin JJ (2015). Survival of patients with stage IV lung cancer with diabetes treated with metformin.. Am. J. Respir. Crit. Care Med..

[37] Li L (2018). Metformin-induced reduction of CD39 and CD73 blocks myeloid-derived suppressor cell activity in patients with ovarian cancer. Cancer Res..

[38] Lapi F (2016). Risk of prostate cancer in low-dose aspirin users: a retrospective cohort study. Int. J. Cancer.

[39] Lee TY (2019). Association of daily aspirin therapy with risk of hepatocellular carcinoma in patients with chronic hepatitis B.. JAMA Intern. Med..

[40] Mikami J (2016). Antitumor effect of antiplatelet agents in gastric cancer cells: an in vivo and in vitro study. Gastric Cancer.

[41] Liang Y (2019). Dichloroacetate overcomes oxaliplatin chemoresistance in colorectal cancer through the miR-543/PTEN/Akt/mTOR pathway. J. Cancer.

[42] Liang Y (2020). MiR-107 confers chemoresistance to colorectal cancer by targeting calcium-binding protein 39. Br. J. Cancer.

[43] Liang Y (2020). Dichloroacetate restores colorectal cancer chemosensitivity through the p53/miR-149-3p/PDK2-mediated glucose metabolic pathway. Oncogene.

[44] Guo F (2019). Inhibitory effect on ovarian cancer ALDH+ stem-like cells by Disulfiram and Copper treatment through ALDH and ROS modulation. Biomed. Pharmacother..

[45] Shah O’Brien P (2019). Disulfiram (Antabuse) activates ROS-dependent ER stress and apoptosis in oral cavity squamous cell carcinoma. J. Clin. Med..

[46] Xu B (2017). Disulfiram/copper selectively eradicates AML leukemia stem cells in vitro and in vivo by simultaneous induction of ROS-JNK and inhibition of NF-kappaB and Nrf2. Cell Death Dis..

[47] Xu Y (2020). Disulfiram/copper markedly induced myeloma cell apoptosis through activation of JNK and intrinsic and extrinsic apoptosis pathways. Biomed. Pharmacother..

[48] Liu P (2012). Cytotoxic effect of disulfiram/copper on human glioblastoma cell lines and ALDH-positive cancer-stem-like cells. Br. J. Cancer.

[49] Pan M (2020). Inhibition of breast cancer growth via miR-7 suppressing ALDH1A3 activity concomitant with decreasing breast cancer stem cell subpopulation. J. Cell Physiol..

[50] DeBerardinis RJ, Chandel NS (2016). Fundamentals of cancer metabolism. Sci. Adv..

[51] Hsu MC, Hung WC (2018). Pyruvate kinase M2 fuels multiple aspects of cancer cells: from cellular metabolism, transcriptional regulation to extracellular signaling. Mol. Cancer.

[52] Zahra K, Dey T, Ashish, Mishra SP, Pandey U (2020). Pyruvate kinase M2 and cancer: the role of PKM2 in promoting tumorigenesis. Front. Oncol..

[53] Chen Z (2015). TRIM35 Interacts with pyruvate kinase isoform M2 to suppress the Warburg effect and tumorigenicity in hepatocellular carcinoma. Oncogene.

[54] Zhou C (2017). RACK1 forms a complex with FGFR1 and PKM2, and stimulates the growth and migration of squamous lung cancer cells. Mol. Carcinog..

[55] Azoitei N (2016). PKM2 promotes tumor angiogenesis by regulating HIF-1alpha through NF-kappaB activation. Mol. Cancer.

[56] Sun X (2019). Intestinal epithelial PKM2 serves as a safeguard against experimental colitis via activating beta-catenin signaling. Mucosal Immunol..

[57] Yang W (2012). ERK1/2-dependent phosphorylation and nuclear translocation of PKM2 promotes the Warburg effect. Nat. Cell Biol..

