# Supplementary material for: Diethyldithiocarbamate-copper complex (CuET) inhibits colorectal cancer progression via miR-16-5p and 15b-5p/ALDH1A3/PKM2 axis-mediated aerobic glycolysis pathway
Source: Oncogenesis. 2021 Jan 8;10(1):4. doi: 10.1038/s41389-020-00295-7 (PMC7794448; doi:10.1038/s41389-020-00295-7)
Supplement: Supplementary file 1 — Supplementary materials and methods [file 41389_2020_295_MOESM1_ESM.docx]

**Supplementary materials and methods**

**Clinical specimen**

The clinical data of 42 CRC patients from the Ninth People’s Hospital Affiliated to Shanghai Jiao Tong University School of Medicine between 2013 and 2017 are shown in **Supplementary Table 1**.

**Chemicals**

All the chemical reagents used in this study are listed in **Supplementary Table 2**.

**Protein extraction and Western blot**

Ripa lysate (Thermo Scientific, USA) containing protease inhibitor cocktail (Kang Cheng, Shanghai, China) was used to lyse cells on ice for 30 minutes. After that, cell lysates were centrifuged at 12,000 g for 20 min, and the supernatant was collected. The protein concentration was determined by plotting standard protein concentration curve using Pierce BCA Protein Assay Kit (Thermo Scientific, USA). The protein was fractionated on 10 or 12% SDS-PAGE and transferred to PVDF membrane (Millipore, USA). After the membrane was sealed with 5% BSA (Bull Serum Albumin) for 2 h, it was incubated overnight at 4°C with the primary antibody. After repeated washing with TBST, the HRP labeled secondary antibody was incubated at room temperature for 1 h. Finally, the ECL detection system (Las 4000 Mini, Japan) was used to detect the signals on the membrane. All the antibodies are shown in **Supplementary Table 2.**

**Cell culture and transfection**

The human colorectal cancer cell lines (HCT116, DLD1, RKO, SW1116, SW480, SW620, Caco2, LoVo and HT29), non-tumorigenic adult human colorectal epithelial cell line (NCM460 and FHC) and normal gastric epithelial cell (GES1) were purchased from American type culture collection (ATCC). The SW480, SW620, RKO, SW1116 and Caco2 cell lines were cultured in Dulbecco’s modified Eagle’s medium (DMEM) with 10% fetal bovine serum (FBS). HCT116 and HT29 cell lines were cultured in McCoy’s 5A medium with 10% FBS. LoVo cells were cultured in F12K medium with 10% FBS. DLD1, NCM460, FHC and GES1 cell lines were cultured in RPMI 1640 medium with 10% FBS. All the cell lines were incubated in a humidified 5% CO2 atmosphere at 37°C.

Cells were transfected with miRNA mimics or miRNA inhibitors or the small interfering RNAs (siRNAs) or their corresponding negative control (NC) that were synthesized and purchased from Genepharm Technologies (Shanghai, China) using DharmaFECT 1 transfection reagent (Thermo Scientific Dharmacon Inc. USA). The plasmids and their negative control were synthesized and purchased from Genechem Co., Ltd (Shanghai, China) and Generay Biotech (Shanghai, China). They were transiently transfected into cells using the FuGENE transfection reagent (Promega, USA). The sequences of miRNA mimics and inhibitors and siRNA involved in the study were listed in **Supplementary Table 3**.

**Cell viability assay**

Cell proliferative ability was evaluated by using cell counting kit-8 (CCK-8; Dojindo, Japan). Briefly, treated and control cells were plated into 96-well plate at 3000 cells per well with the corresponding medium. 10 μl CCK-8 and 90 μl culture medium were added to each well at specified time points. After incubating at 37°C for another 2 h, cell viability was determined by measuring the optical density (OD) at 450 nm with a microplate reader (BioTeck, Vermont, USA). The inhibition ratio was calculated with the following formula: [(Ac-As)/(Ac-Ab)] × 100%. The IC50 of each cell line was calculated by GraphPad Prism 7 (GraphPad Software, San Diego, CA).

**Flow cytometry analysis**

For cell cycle analysis, exponentially growing cells were treated for 24 h. Cells were then collected and fixed overnight with ice-cold 75% ethanol at 4°C. Cells were then washed twice with PBS, and resuspended in PI/RNase staining buffer (BD, CA, USA). The samples were analyzed by flow cytometry (BD, FACSCalibur).

Cells with various treatments were collected and stained with Annexin V-FITC and PI. Apoptosis was detected using the BD Pharmingen Kit (BD, CA, USA) according to the manufacturer's protocol and analyzed by flow cytometry (BD, FACSCalibur).

**Colony formation assay**

The treated cells were seeded into six-well plates at 1000 cells per well and cultured in fresh medium at 37°C for 2 weeks. The cell colonies were fixed with 4% paraformaldehyde for 30 min and stained with 1% crystal violet. Finally, the number of colonies was counted by a counter (Gelcount, Optronix, Oxford).

**Dual-luciferase activity reporter assay**

A transient transactivation assay was performed using ALDH1A3 promoter to test whether CuET regulates ALDH1A3 at transcriptional level. To this end, CRC cells treated with CuET or DMSO were seeded into 24-well plates, co-transfected with 20 ng Renilla and 200 ng ALDH1A3-GV238, a luciferase reporter driven by ALDH1A3 promoter or the GV238-Basic vector with FuGene transfection reagent. 24 hours later, luciferase activity was analyzed using a luciferase assay kit (Promega, Madison, USA). Each transfection was performed in three times and repeated twice.

ALDH1A3 mRNA 3’-untranslated region (3’-UTR) containing the putative miR-16-5p and miR-15b-5p binding sites was cloned into Firefly/Renilla Dual-Luciferase plasmid. The mutant binding sequences were inserted into an identical Luciferase plasmid to prove the binding specificity. Cells were seeded in 24 well plates and co-transfected with firefly luciferase reporter plasmid, Renilla plasmid, miR-16-5p (50 pmol) or miR-15b-5p (50 pmol), miR-16-5p and miR-15b-5p (25 pmol each) mimics or negative control miRNA. Twenty-four hours after transfection, luciferase activity was analyzed using a luciferase assay kit (Promega, Madison, USA). Each transfection was performed in triplicate and repeated twice.

**RNA extraction and quantitative real-time PCR**

Total RNA was extracted from CRC tissues or cultured cells with TrIzol reagent (Life, CA, USA). cDNA was reverse-transcribed using the PrimeScript RT Reagent Kit (TaKaRa, Tokyo, Japan). Quantitative real-time PCR was performed on ABI-7500 platform (Applied Biosystems, Grand Island, NY, USA) using premix Ex Taq 420 A (TaKaRa, Tokyo, Japan). Ct values for mRNA and miRNA were normalized to actin and U6 as endogenous controls. The relative expressions of target genes were quantified by the 2−ΔΔCt method. All primer sequences are shown in **Supplementary Table 4**.

**High-Throughput RNA-seq analysis**

High-Throughput RNA-seq analysis was performed by OE Biotechnology Co., Ltd. (Shanghai, China). Briefly, RNAs were isolated separately from four pairs of HCT116 cells treated with 1.0 μM CuET and DMSO treated as control by using the TrIzol reagent. The quality and concentration of each sample were analyzed on an Agilent 2100 Bioanalyzer. The libraries were sequenced on an Illumina HiSeq 3000 platform.

**Immunohistochemistry**

Colorectal cancer and normal tissue sections were stained with primary antibodies against ALDH1A3 (Abcam, USA). The tissue slides were evaluated by two investigators independently. Protein expression was assessed on the basis of the intensity (the grade was measured on a scale of 0-3: 0, no staining; 1, weak staining; 2, moderate staining; 3, strong staining) and extent of staining (the percentage of positive tumor cells was measured on a scale of 0-4: 0, none; 1, 1-25%; 2, 26-50%; 3, 51-75%; 4, >75%). To obtain the final score, we multiplied the extent by grades of intensity staining. Then the samples were ranked by the final score. Ultimately, the protein expression was sorted into high expression and low expression by the median of the sample size.

**Lactate, ATP and glucose uptake assays**

Cells were seeded into a six-well plate, treated with CuET or transfected with miR mixture mimics, miR mixture inhibitors, PKM2 siRNA, ALDH1A3 overexpressing plasmid and their corresponding control. Lactate product assays were performed using a Lactate Colorimetric Assay Kit (ab65331 Abcam). ATP levels were measured using an ATP assay kit (ab83355 Abcam). Glucose uptake was detected using Glucose Uptake Assay Kit (Colorimetric) (ab136955, Abcam). All experiments were performed according to the corresponding manufacturer's instructions. Data were normalized to total cell number.

**Seahorse metabolic analysis**

ECAR and OCR were measured using Seahorse XF Glycolysis Stress Test Kit and Seahorse XF Cell Mito Stress Test Kit (Agilent Technologies, Palo Alto, CA). CRC cells were seeded into the 96-well cell culture plates in medium with 10% FBS and incubated at 37℃ overnight and then the cells were used for measurement of ECAR and OCR. After measurement of baseline concentration, glucose, oligomycin, and 2-DG were sequentially added into each well for ECAR measurement. Oligomycin, FCCP (p-trifluoromethoxy carbonyl cyanide phenylhydrazone), and Antimycin A & Rotenone were sequentially injected into each well for OCR measurement. Seahorse XF-96 Wave software was used to analyze the data.

**Co-immunoprecipitation (Co-IP) and mass** **spectrometry**

To detect the protein-protein interaction, the soluble proteins were extracted from the transfected cells with the Pierce IP lysis buffer (Thermo Fisher Science) added with protease inhibitor cocktail and 1 mM DTT. After incubation with anti-Flag or anti-Myc antibody or anti-ALDH1A3 or anti-PKM2 antibody or control IgG overnight, 10 µl Protein G sepharose beads (Thermo Fisher Science) were added and the mixture was incubated at 4°C for another 2 h. The samples were then washed with IP lysis buffer three or four times. The immobilized protein complex was denatured at 100°C for 10 min in 2 × SDS sample buffer and then Western blotting was performed. Candidate bands were excised and subjected to trypsin digestion. Liquid chromatography tandem mass spectrometry (LC-MS) analyses of protein digests were carried out on a mass spectrometer (LTQ Obitrap ETD, Thermo Scientific) coupled with liquid chromatography (EASY-nLC 1000, Thermo Scientific) for protein identification.

**Ubiquitination assay**

HCT116 and LoVo cells transfected with Flag-tagged ALDH1A3 overexpressed plasmid or empty vector and treated with 20 μM MG132 (Sigma-Aldrich, St. Louis, MO, USA) for 6 h. Total proteins were extracted using the Pierce IP lysis buffer supplemented with proteinase inhibitor. Immunoprecipitation was then performed using anti-PKM2 or anti-IgG, respectively. The ubiquitination level of the immunoprecipitated protein was evaluated by Western blotting using anti-ubiquitin. The inputs were subjected to Western blot analysis with antibodies against ALDH1A3, PKM2 and β-actin, respectively.

**Statistics**

All data were presented as Mean ± SD. All *in vitro* experiments were conducted in triplicate. IC50 values were calculated and statistical analyses were performed using Microsoft Excel and GraphPad Prism Software version 7.0. To compare experimental data of two groups, a two-tailed student t-test was used. To compare results of more than two groups, one-way analysis of variance (ANOVA) followed by Dunnett’s test was carried out. A Log-rank test was used to assess the Kaplan–Meier curves. The Cox univariate proportional hazards regression model was used to determine the independent clinical factors based on the investigated variables. Spearman’s correlation was performed to analyze the correlation between microRNA and ALDH1A3. *p* < 0.05 was considered statistically significant.
