# Supplementary material for: Diethyldithiocarbamate-copper complex (CuET) inhibits colorectal cancer progression via miR-16-5p and 15b-5p/ALDH1A3/PKM2 axis-mediated aerobic glycolysis pathway
Source: Oncogenesis. 2021 Jan 8;10(1):4. doi: 10.1038/s41389-020-00295-7 (PMC7794448; doi:10.1038/s41389-020-00295-7)
Supplement: Supplementary file 2 — Supplementary Table1-6 [file 41389_2020_295_MOESM2_ESM.docx]

**Supplementary Table 1: Characteristics of CRC patients**

| **Characteristic** | **Total (n=42)** |
| --- | --- |
| Mean age (years ± SD) | 59.8 ± 12.3 |
| Gender |  |
| Male | 31 (73.8%) |
| Female | 11 (26.2%) |
| Localization |  |
| Rectum | 13 (31.0%) |
| Colon | 29 (69.0%) |
| Tumor size |  |
| ≤4cm | 24 (57.1%) |
| > 4cm | 18 (42.9%) |
| T classification |  |
| T1/T2 | 15 (35.7%) |
| T3/T4 | 27 (64.3%) |
| Lymph nodes metastasis |  |
| N0 | 12 (28.6%) |
| N1/N2 | 30 (71.4%) |
| Distant metastasis |  |
| M0 | 38 (90.5%) |
| M1 | 4 (9.5%) |
| Histological differentiation |  |
| High/moderate | 32 (76.2%) |
| Low | 10 (23.8%) |

**Supplementary Table 2: Antibodies and chemical reagents used in this study**

| **Primary Antibodies** | **Source** | **identifier** |
| --- | --- | --- |
| Apoptosis Antibody Sampler Kit | Cell Signaling Technology | Cat#9915 |
| Cell Cycle Regulation II Antibody Sampler Kit | Cell Signaling Technology | Cat #9870 |
| Anti-ALDH1A3 | Abcam | Cat #ab129815 |
| Anti-PKM2 | Santa Cruz Biotechnology | Cat #sc-365684 |
| Anti-FLAG | Sigma-Aldrich | Cat #F1804-200UG |
| Anti-FLAG | Cell Signaling Technology | Cat #15010 |
| Anti-Myc | Cell Signaling Technology | Cat #2276s |
| Anti-GAPDH | Proteintech | Cat # 60004-1-Ig |
| Anti-β-Actin-HRP | Kangcheng | Cat # KC-5A08 |
| Normal Rabbit IgG | Cell Signaling Technology | Cat #2729S |
| [Normal Mouse IgG](https://www.scbt.com/zh/p/normal-mouse-igg?requestFrom=search) | Santa Cruz Biotechnology | Cat #sc-2025 |
| Anti-Ubiquitin | Cell Signaling Technology | Cat # 3936s |
| Anti-Bax | Abcam | Cat #ab32503 |
| Anti-Bcl-2 | Abcam | Cat #ab182858 |
| Anti- Cleaved Caspase3 | Abcam | Cat #ab2302 |
| Anti-GAPDH | Cell Signaling Technology | Cat #5174 |
| **Secondary Antibodies** |  |  |
| Goat anti-Rabbit IgG antibody (HRP) | Arigo Biolaboratories | Cat # ARG65351 |
| Goat anti-Mouse IgG antibody (HRP) | Arigo Biolaboratories | Cat # ARG65350 |
| Goat Anti-Mouse IgG Light Chain Specific | Jackson | Cat # 115-035-174 |
| Mouse Anti-Rabbit IgG Light Chain | Abcam | Cat #Ab99697 |
| Goat anti-Rabbit IgG antibody (HRP) | [Beyotime Biotechnology](http://www.baidu.com/link?url=iZdendGs4P1EkDNTjQBNZYsw3SkB8A9Rx_tu_641QKyzJU-NNBnYd8pd6EzuYK_K) | Cat #A0208 |
| Goat anti-Mouse IgG antibody (HRP) | [Beyotime Biotechnology](http://www.baidu.com/link?url=iZdendGs4P1EkDNTjQBNZYsw3SkB8A9Rx_tu_641QKyzJU-NNBnYd8pd6EzuYK_K) | Cat #A0216 |
| **Chemicals** |  |  |
| \|  \| Diethyldithiocarbamic acid sodium salt \| \| --- \| --- \| | Sangon Biotech | Cat #A601356 |
| Copper (II) chloride | Sangon Biotech | Cat #A603090 |
| DTT | Sigma-Aldrich | Cat #43819 |
| CHX | Sigma-Aldrich | Cat # 5.08739 |
| MG132 | Sigma-Aldrich | Cat # M8699 |

**Supplementary Table 3: The Sequences for RNA Oligos**

| **RNA Oligo Names** | **sense (5’-3’)** | **antisense (5’-3’)** |
| --- | --- | --- |
| hsa-miR-16-5p mimics | UAGCAGCACGUAAAUAUUGGCG | CCAAUAUUUACGUGCUGCUAUU |
| hsa-miR-15b-5p mimics | UAGCAGCACAUCAUGGUUUACA | UAAACCAUGAUGUGCUGCUAUU |
| Negative control | UUCUCCGAACGUGUCACGUTT | ACGUGACACGUUCGGAGAATT |
| hsa-miR-16-5p inhibitor | CGCCAAUAUUUACGUGCUGCUA |  |
| hsa-miR-15b-5p inhibitor | UGUAAACCAUGAUGUGCUGCUA |  |
| miRNA inhibitor N.C. | CAGUACUUUUGUGUAGUACAA |  |
| ALDH1A3-siRNA1 | GUCAAGUUCACCAAGAUAUTT | AUAUCUUGGUGAACUUGACTT |
| ALDH1A3-siRNA2 | GGGCCUCAGAUUGAUCAAATT | UUUGAUCAAUCUGAGGCCCTT |
| PKM2-siRNA | GCUGUGGCUCUAGACACUATT | UAGUGUCUAGAGCCACAGCTT |

**Supplementary Table 4: Primer sequences used in this study**

| ALDH18A | Forward | 5'- TGAGCTGAAGCATGCCAAGA -3’ |
| --- | --- | --- |
|  | Reverse | 5'- CACATTCATCCCCTCGGGTC -3’ |
| ALDH1A3 | Forward | 5'- GATGCCGCCACTCGCTACATAG -3’ |
|  | Reverse | 5'- TAGACCTGCTCCTCCACGAACAC -3’ |
| ALDH1B1 | Forward | 5'- CGGGATCGAGTCTACTTGGC -3’ |
|  | Reverse | 5'- GGAAAGCCTGCCTCCTTGAT -3’ |
| ALDH3B1 | Forward | 5'- AAACAAGCAGCTTCTGCACG -3’ |
|  | Reverse | 5'- CAGGGCTGCATAGGACGTAG -3’ |
| ALDH5A1 | Forward | 5'-GAGAGGAGTTCATTACTTCGGA -3’ |
|  | Reverse | 5'-GCTGTGATTATTCTGGCAAGGT -3’ |
| ALDOA | Forward | 5’-AAGGCTGCTCCATCAACACTC -3’ |
|  | Reverse | 5’-ATTCACAGACAACACCGCACA -3’ |
| ENO1 | Forward | 5’-TGGGAAAGCTGGCTACACTG -3’ |
|  | Reverse | 5’- CTGGTCAGGCGAGATGTACC -3’ |
| PKM2 | Forward | 5'-ACTCGGGCTGAAGGCAGTGA -3’ |
|  | Reverse | 5'-TGTGGGGTCGCTGGTAATGG -3’ |
| LDHA | Forward | 5'- TGGCAGATGAACTTGCTCTTG -3’ |
|  | Reverse | 5'- GGAAAAGGCTGCCATGTTGG -3’ |
| PDK1 | Forward | 5'- GCTGTATGGCCTGCAAGATG -3’ |
|  | Reverse | 5'- GCTGTCCTGGTGATTTTGCA -3’ |
| Actin | Forward | 5’- CTCCATCCTGGCCTCGCTGT -3’ |
|  | Reverse | 5’- GCTGTCACCTTCACCGTTCC -3’ |
| miR-497-5p | RT | 5’-GTCGTATCCAGTGCAGGGTCCGAGGTATTCGCACTGGATACGACACAAAC -3’ |
|  | Forward | 5’-GCGCAGCAGCACACTGTG -3’ |
|  | Reverse | 5’-AGTGCAGGGTCCGAGGTATT -3’ |
| miR-16-5p | RT | 5’-GTCGTATCCAGTGCAGGGTCCGAGGTATTCGCACTGGATACGACCGCCAA -3’ |
|  | Forward | 5’- CGCGTAGCAGCACGTAAATA -3’ |
|  | Reverse | 5’- AGTGCAGGGTCCGAGGTATT -3’ |
| miR-200c-3p | RT | 5’-GTCGTATCCAGTGCAGGGTCCGAGGTATTCGCACTGGATACGACTCCATC -3’ |
|  | Forward | 5’-CGCGTAATACTGCCGGGTAAT -3’ |
|  | Reverse | 5’-AGTGCAGGGTCCGAGGTATT -3’ |
| miR-200b-3p | RT | 5’-GTCGTATCCAGTGCAGGGTCCGAGGTATTCGCACTGGATACGACTCATCA -3’ |
|  | Forward | 5’-GCGCGTAATACTGCCTGGTAA -3’ |
|  | Reverse | 5’-AGTGCAGGGTCCGAGGTATT -3’ |
| miR-15a-5p | RT | 5’-GTCGTATCCAGTGCAGGGTCCGAGGTATTCGCACTGGATACGACCACAAA -3’ |
|  | Forward | 5’-CGCGTAGCAGCACATAATGG -3’ |
|  | Reverse | 5’-AGTGCAGGGTCCGAGGTATT -3’ |
| miR-429 | RT | 5’-GTCGTATCCAGTGCAGGGTCCGAGGTATTCGCACTGGATACGACACGGTT -3’ |
|  | Forward | 5’-CGCGCGTAATACTGTCTGGTAA -3’ |
|  | Reverse | 5’-AGTGCAGGGTCCGAGGTATT -3’ |
| miR-195-5p | RT | 5’-GTCGTATCCAGTGCAGGGTCCGAGGTATTCGCACTGGATACGACGCCAAT -3’ |
|  | Forward | 5’-GCGCGTAGCAGCACAGAAAT -3’ |
|  | Reverse | 5’-AGTGCAGGGTCCGAGGTATT -3’ |
| miR-424-5p | RT | 5’-GTCGTATCCAGTGCAGGGTCCGAGGTATTCGCACTGGATACGACTTCAAA -3’ |
|  | Forward | 5’-GCGCAGCAGCAATTCATGT -3’ |
|  | Reverse | 5’-AGTGCAGGGTCCGAGGTATT -3’ |
| miR-15b-5p | RT | 5’-GTCGTATCCAGTGCAGGGTCCGAGGTATTCGCACTGGATACGACTGTAAA -3’ |
|  | Forward | 5’-CGCGTAGCAGCACATCATGG -3’ |
|  | Reverse | 5’-AGTGCAGGGTCCGAGGTATT -3’ |
| miR-329-3p | RT | 5’-GTCGTATCCAGTGCAGGGTCCGAGGTATTCGCACTGGATACGACAAAGAG -3’ |
|  | Forward | 5’-CGCGAACACACCTGGTTAAC -3’ |
|  | Reverse | 5’-AGTGCAGGGTCCGAGGTATT -3’ |
| miR-8485 | RT | 5’-GTCGTATCCAGTGCAGGGTCCGAGGTATTCGCACTGGATACGACATACGT -3’ |
|  | Forward | 5’-CGCGCACACACACACACAC -3’ |
|  | Reverse | 5’-AGTGCAGGGTCCGAGGTATT -3’ |
| miR-6858-5p | RT | 5’-GTCGTATCCAGTGCAGGGTCCGAGGTATTCGCACTGGATACGACGTCCCT -3’ |
|  | Forward | 5’-GGTGAGGAGGGGCTGGC -3’ |
|  | Reverse | 5’-AGTGCAGGGTCCGAGGTATT -3’ |
| miR-4689 | RT | 5’-GTCGTATCCAGTGCAGGGTCCGAGGTATTCGCACTGGATACGACGGCCCC -3’ |
|  | Forward | 5’-CGCGTTGAGGAGACATGGTG -3’ |
|  | Reverse | 5’-AGTGCAGGGTCCGAGGTATT -3’ |
| miR-34b-5p | RT | 5’-GTCGTATCCAGTGCAGGGTCCGAGGTATTCGCACTGGATACGACCAATCA -3’ |
|  | Forward | 5’-CGCGTAGGCAGTGTCATTAGC -3’ |
|  | Reverse | 5’-AGTGCAGGGTCCGAGGTATT -3’ |
| miR-7-5p | RT | 5’-GTCGTATCCAGTGCAGGGTCCGAGGTATTCGCACTGGATACGACAACAAC -3’ |
|  | Forward | 5’-CGCGTGGAAGACTAGTGATTTT -3’ |
|  | Reverse | 5’-AGTGCAGGGTCCGAGGTATT -3’ |
| U6 | Forward | 5’-GCTTCGGCAGCACATATACTAAAAT -3’ |
|  | RT (Reverse) | 5’-CGCTTCACGAATTTGCGTGTCAT -3’ |

**Supplementary Table 5: miRNAs were predicted in greater than or equal to three databases overlap.**

| **databases** | **miRNAs** |
| --- | --- |
| RNA22, TargetScan, miRDB, miRanda | hsa-miR-16-5p |
| TargetScan, miRDB, miRanda | hsa-miR-497-5p |
|  | hsa-miR-200c-3p |
|  | hsa-miR-200b-3p |
|  | hsa-miR-15a-5p |
|  | hsa-miR-429 |
|  | hsa-miR-195-5p |
|  | hsa-miR-424-5p |
|  | hsa-miR-15b-5p |
| TargetScan, miRTarBase, miRanda | hsa-miR-329-3p |
| TargetScan, miRDB, miRTarBase | hsa-miR-8485 |
|  | hsa-miR-6858-5p |
|  | hsa-miR-4689 |
| RNA22, TargetScan, miRDB | hsa-miR-34b-5p |
| RNA22, TargetScan, miRTarBase | hsa-miR-7-5p |

**Supplementary Table 6: List of potential target genes of ALDH1A3 identified by LC-MS/MS.**

| **IP: IgG** | | **IP: ALDH1A3** | |
| --- | --- | --- | --- |
| **Gene Name** | **Score** | **Gene Name** | **Score** |
| HSP90AB1 | 10.83 | HSPA9 | 55.66 |
| CNBP | 6.81 | CNBP | 32.03 |
| HSPA8 | 9.39 | HSPA8 | 56.7 |
| HSPA9 | 12.40 | HSP90AB1 | 14.07 |
| DCBLD1 | 0.00 | HSPD1 | 22.35 |
| HSPA6 | 2.66 | RPL4 | 8.15 |
| NAV2 | 0.00 | HSPA5 | 20.55 |
| CCT3 | 0.00 | ACTB | 39.39 |
| CCT8 | 0.00 | ALDH1A3 | 56.3 |
| TUBA1A | 1.80 | ENO1 | 17.83 |
| TUBB | 3.77 | ANXA2 | 35.34 |
|  |  | TRIM21 | 21.57 |
|  |  | ALDOA | 21.74 |
|  |  | HSPA1A | 31.31 |
|  |  | HSP90AA1 | 14.85 |
|  |  | PPIA | 20.54 |
|  |  | LMNA | 12.87 |
|  |  | PKM2 | 19.88 |
|  |  | TCP1 | 22.39 |
|  |  | CCT4 | 19.21 |
|  |  | CCT5 | 8.24 |
|  |  | CCT8 | 15.47 |
|  |  | TUBA1A | 33.95 |
|  |  | TUBA1B | 36.34 |
|  |  | TUBA4A | 26.07 |
|  |  | TUBB | 61.62 |
|  |  | TUBB4B | 56.93 |
|  |  | TUBB6 | 26.43 |
