# Supplementary figures and images for: Diethyldithiocarbamate-copper complex (CuET) inhibits colorectal cancer progression via miR-16-5p and 15b-5p/ALDH1A3/PKM2 axis-mediated aerobic glycolysis pathway
Source: Oncogenesis. 2021 Jan 8;10(1):4. doi: 10.1038/s41389-020-00295-7 (PMC7794448; doi:10.1038/s41389-020-00295-7)

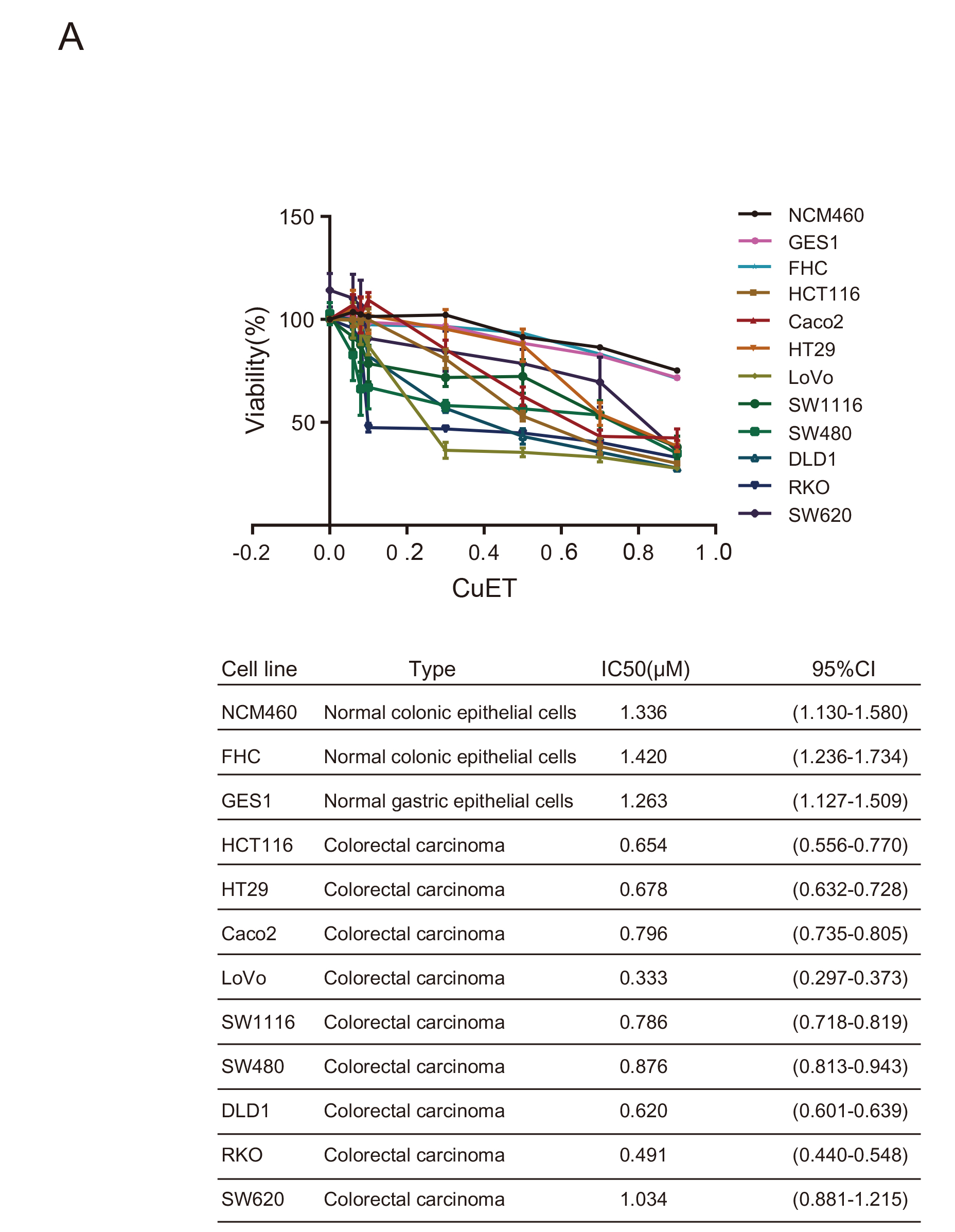

Supplement: Supplementary file 3 — Supplementary Figure 1 [file 41389_2020_295_MOESM3_ESM.jpg]

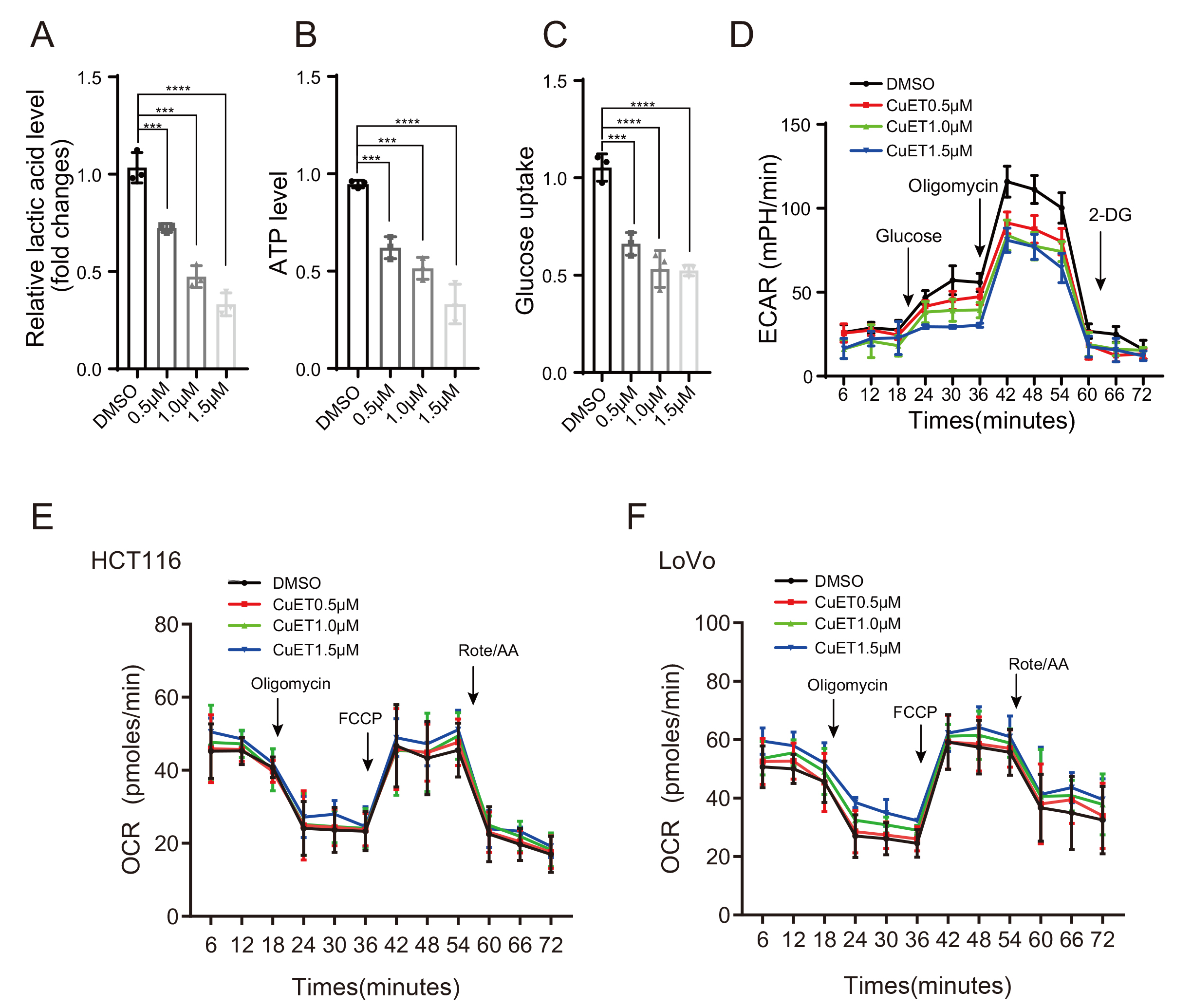

Supplement: Supplementary file 4 — Supplementary Figure 2 [file 41389_2020_295_MOESM4_ESM.jpg]

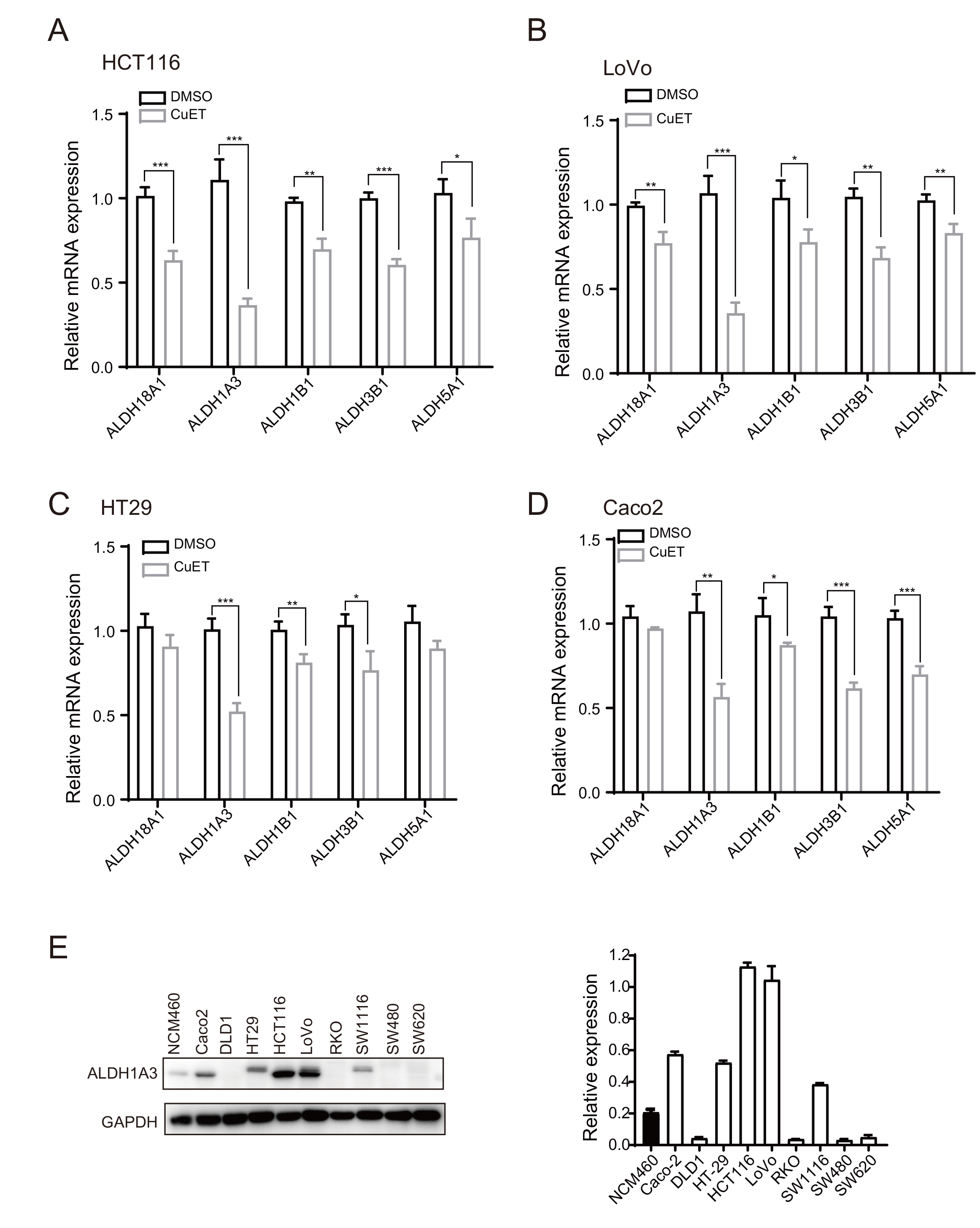

Supplement: Supplementary file 5 — Supplementary Figure 3 [file 41389_2020_295_MOESM5_ESM.jpg]

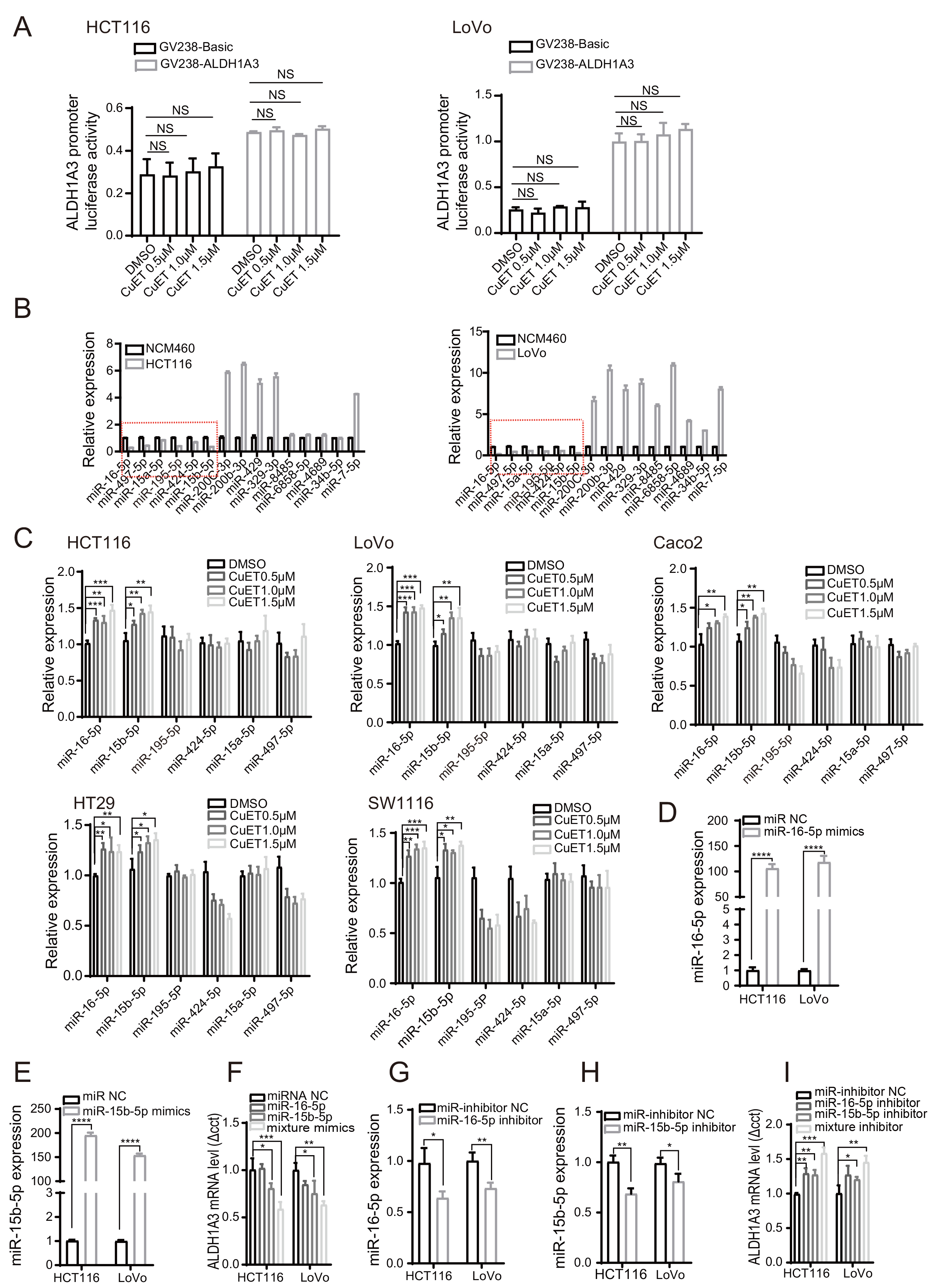

Supplement: Supplementary file 6 — Supplementary Figure 4 [file 41389_2020_295_MOESM6_ESM.jpg]

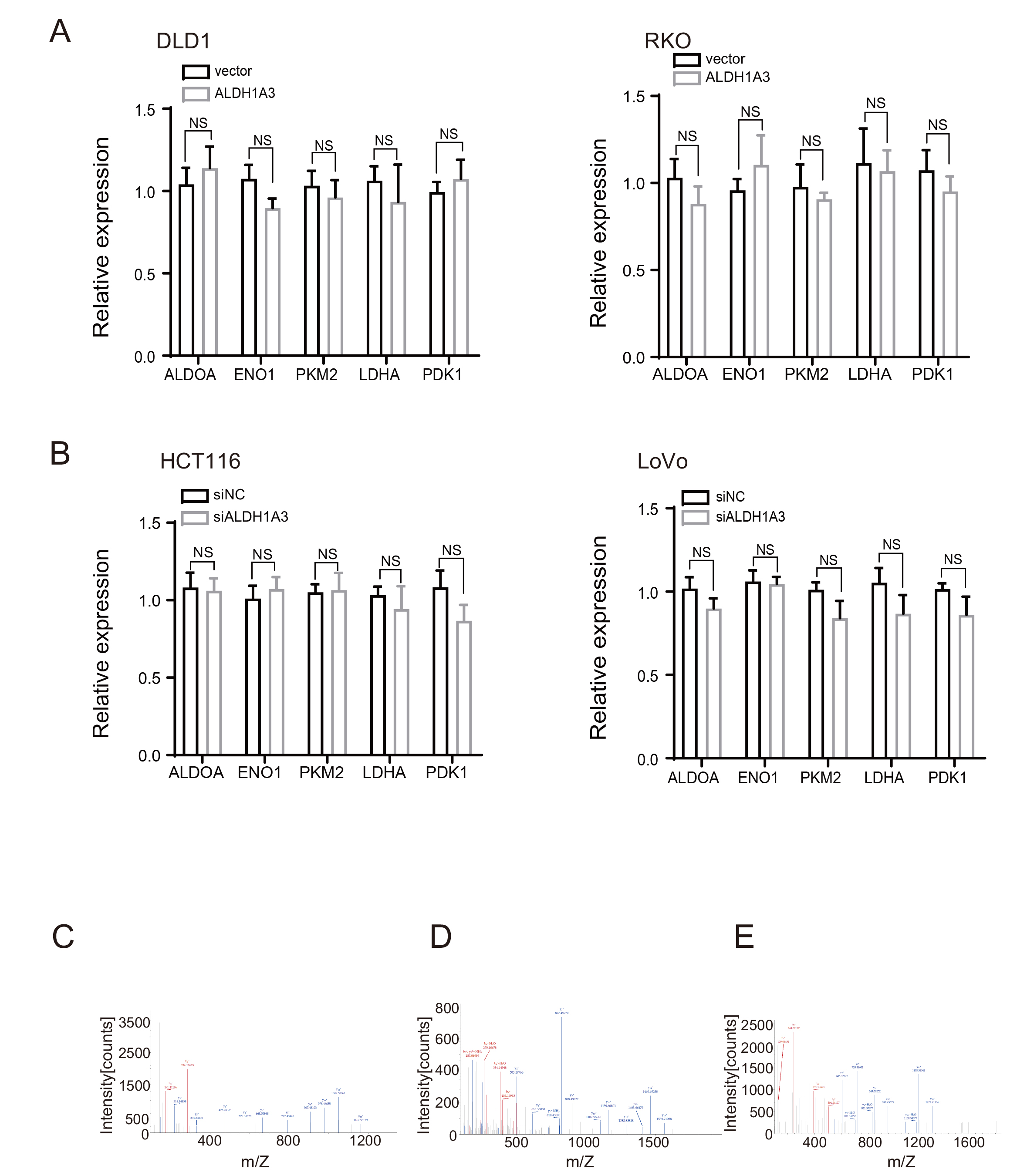

Supplement: Supplementary file 7 — Supplementary Figure 5 [file 41389_2020_295_MOESM7_ESM.jpg]

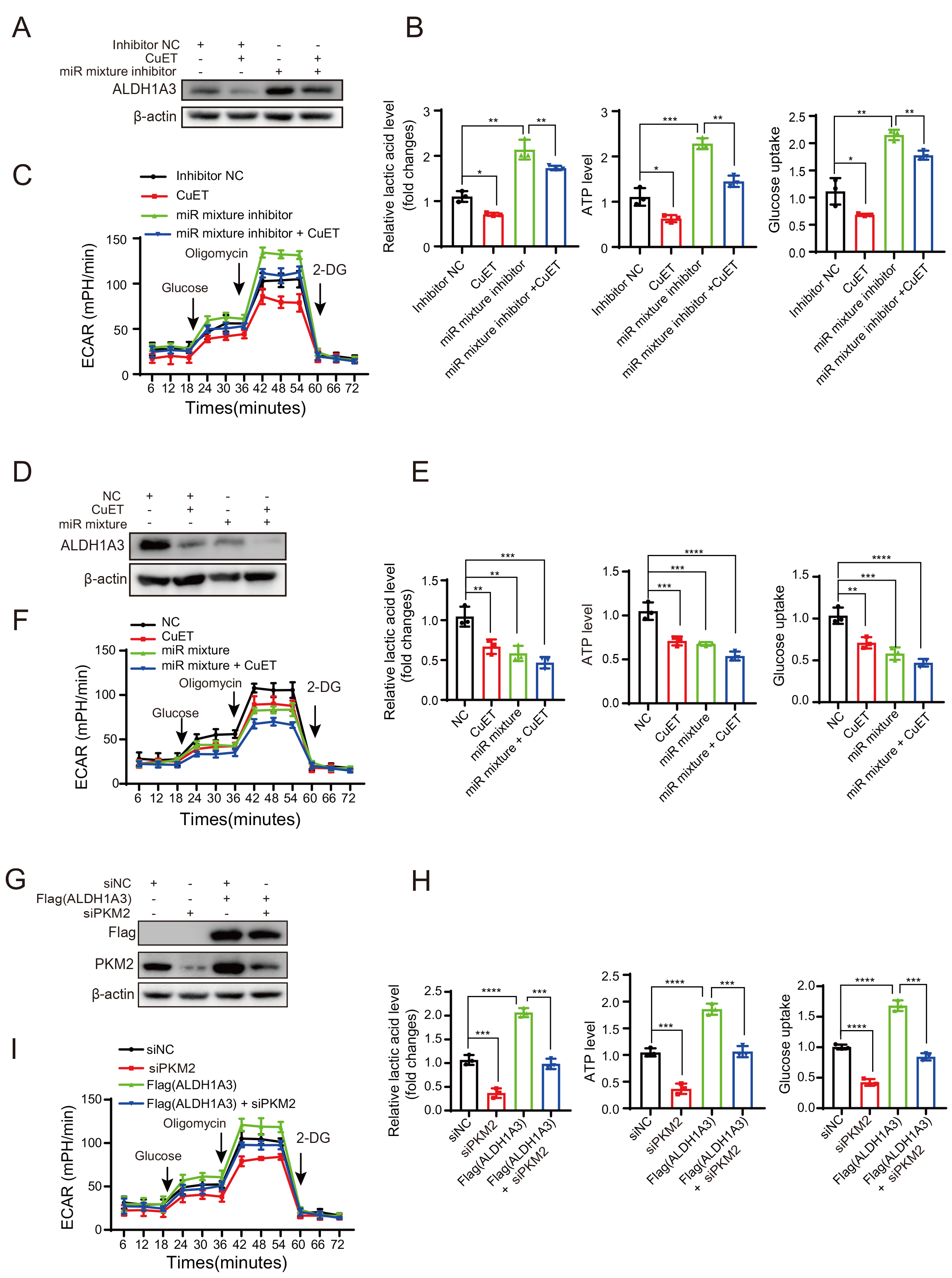

Supplement: Supplementary file 8 — Supplementary Figure 6 [file 41389_2020_295_MOESM8_ESM.jpg]
