# Supplementary material for: Diethyldithiocarbamate-copper complex (CuET) inhibits colorectal cancer progression via miR-16-5p and 15b-5p/ALDH1A3/PKM2 axis-mediated aerobic glycolysis pathway
Source: Oncogenesis. 2021 Jan 8;10(1):4. doi: 10.1038/s41389-020-00295-7 (PMC7794448; doi:10.1038/s41389-020-00295-7)
Supplement: Supplementary file 9 — Supplementary figure legends [file 41389_2020_295_MOESM9_ESM.docx]

**Supplementary Figure Legends**

**Supplementary Fig. S1 The effects of CuET on CRC and non-tumor cell lines. a** A panel of CRC, a normal gastric epithelial cell line and two normal colorectal epithelial cells were treated with CuET at the indicated concentrations for 24 h, and cell viabilities were measured by CCK8 assay. IC50 values of each cell line documented differential CuET induced cytotoxicity (24h treatment).

**Supplementary Fig. S2 CuET attenuates Warburg effect in CRC cells. a-c** Extracellular lactate production **(a)**, cellular ATP level **(b)** and cellular glucose uptake **(c)** were measured in LoVo cells treated with CuET. **(d)** Extracellular acid ratio (ECAR) was determined in LoVo cells treated with different concentrations of CuET. Data are presented as mean ± SD of three independent experiments, ****p*<0.001, *****p*<0.0001. **e, f** Oxygen consumption rate (OCR) was determined in HCT116 **(e)** and LoVo **(f)** cells treated with different concentrations of CuET. Data are presented as mean ± SD of three independent experiments, *p*$>$0.05.

**Supplementary Fig.** **S3** **ALDH1A3 is the potential target gene of CuET. a-d** CRC cells were treated with DMSO or CuET 1.0 μM, and Real-time PCR was performed to detect ALDH18A1, ALDH1A3, ALDH1B1, ALDH3B1 and ALDH5A1 expression in HCT116 (**a**), LoVo (**b**), HT29 (**c**) and Caco2 (**d**). **e** Western blot of ALDH1A3 from CRC cell lines and NCM460 cells (left panel), and their relative expression was shown in the right panel. Data are presented as mean ± SD of three independent experiments, **p*<0.05, ***p*<0.01, ****p*<0.001.

**Supplementary Fig. S4** **CuET inhibits ALDH1A3 by selectively enhancing expressions of miR-16-5p and miR-15b-5p. a** Luciferase assays were performed in HCT116 and LoVo cells. Cells were treated with CuET or DMSO after transfection of ALDH1A3 promoter plasmids. NS, not significant, *p*>0.05. **b** The expression of 15 candidate miRNAs was detected by qPCR. 6 miRNAs in HCT116 and LoVo cells were significantly downregulated compared with NCM460 cells that were shown in the red box (*p*<0.05). **c** miR-16-5p and miR-15b-5p were the most significantly upregulated in response to CuET intervention in HCT116, LoVo, Caco2, HT29 and SW1116 cells. **d** Real-time PCR showed relative expression of miR-16-5p in CRC cells transfected with miR-16-5p mimics. **e** Real-time PCR showed relative expression of miR-15b-5p in CRC cells transfected with miR-15b-5p mimic. **f** Real-time PCR showed relative expression of ALDH1A3 in CRC cells transfected with miR-16-5p, miR-15b-5p or miR-16-5p/15b-5p mixture mimics. **g** Real-time PCR showed relative expression of miR-16-5p in CRC cells transfected with miR-16-5p inhibitor. **h** Real-time PCR showed relative expression of miR-15b-5p in CRC cells transfected with miR-15b-5p inhibitor. **i** Real-time PCR showed relative expression of ALDH1A3 in CRC cells transfected with miR-16-5p, miR-15b-5p or miR-16-5p/15b-5p mixture inhibitors. Data of three independent experiments are shown as means ± SD, **p*<0.05, ***p*<0.01, ****p*<0.001.

**Supplementary Fig. S5 Effect of ALDH1A3 on mRNA expression of genes related to glycolysis and the identification of possible downstream genes of ALDHA1A3. a** Real-time PCR showed the mRNA levels of 5 candidate genes related to glycolysis in DLD1 and RKO cells transfected with ALDH1A3 plasmid. **b** Real-time PCR showed the mRNA levels of 5 candidate genes related to glycolysis in HCT116 and LoVo cells transfected with siALDH1A3. Data are presented as mean ± SD of three independent experiments, *p*$>$0.05. **c-e** Peptides of ALDOA **(c)**, ENO1 **(d)** and PKM2 **(e)** binding to ALDH1A3 were shown in the MS/MS spectrum.

**Supplementary Fig. S6 miR-16-5p, miR-15b-5p/ALDH1A3 axis regulates glycolysis *in vivo*. a** Western blot showed the expression of ALDH1A3 in LoVo cells treated with CuET, transfected with inhibitor NC, mixture inhibitors or mixture inhibitors plus CuET treatment. **b, c** Their lactate production, ATP level, glucose uptake **(b)** and ECAR **(c)** were determined respectively. **d** Western blot showed the expression of ALDH1A3 in LoVo cells treated with CuET, transfected with NC, mixture mimics or mixture mimics plus CuET treatment. **e, f** Their lactate production, ATP level, glucose uptake **(e)** and ECAR **(f)** were determined respectively. **g** Western blot showed the expression of PKM2 in LoVo cells transfected with siNC, PKM2 siRNA, Flag-tagged ALDH1A3 plasmid or PKM2 siRNA plus ALDH1A3 plasmid. **h, i** Their lactate production, ATP level, glucose uptake **(h)** and ECAR **(i)** were determined respectively. Data shown are mean ± SD of three independent experiments,, ***p*<0.01, ****p*<0.001, *****p*<0.0001.
